# Cellular localization of a variant RAPGEF5 protein associated with idiopathic epilepsy risk in the Belgian shepherd

**DOI:** 10.1186/s40575-024-00138-3

**Published:** 2024-09-29

**Authors:** Dawn D. Cayabyab, Janelle M. Belanger, Claudia Xu, Elizabeth A. Maga, Anita M. Oberbauer

**Affiliations:** Department of Animal Science, University of California, One Shields Ave, Davis, CA 95616 USA

**Keywords:** Idiopathic epilepsy, Belgian shepherd, Dog, *RAPGEF5*, Cell localization, Wnt signaling

## Abstract

The Wnt signaling pathway is critical for normal embryonic development. Disruptions in the Wnt signaling pathway have been linked to neurological disorders. The RAPGEF5 protein is a partner in Wnt signaling and a *RAPGEF5* 3-bp insertion is associated with increased risk for idiopathic epilepsy in the Belgian shepherd dog. The 3-bp insertion risk variant introduces an alanine residue predicted to disrupt the protein. Wildtype and the risk variant RAPGEF5 cDNAs were cloned into green fluorescent protein (GFP) expression vectors and transfected into canine kidney cells. The cellular localization of each GFP-labeled RAPGEF5 protein was assessed. Variant RAPGEF5 protein was altered in its localization from that of the wildtype protein and rather than localized to the nucleus and cytoplasm as seen for the wildtype, it was predominantly found in the cytoplasm. Belgian shepherds with the risk variant for RAPGEF5 may have altered Wnt signaling due to modified intracellular localization which in turn could thereby contribute to the expression of idiopathic epilepsy.

**Supplementary Information:**

The online version contains supplementary material available at 10.1186/s40575-024-00138-3.

## Abstract (plain language summary)

A small insertion in the *RAPGEF5* gene is shown to be associated with an increased risk for idiopathic epilepsy in Belgian Shepherds. The insertion introduces an additional amino acid that is predicted to disrupt the RAPGEF5 protein. This protein is involved in the Wnt signaling pathway which is critical for normal embryonic development and disturbances in the Wnt pathway have been linked to neurological disorders. To determine the impact of the insertion on *RAPGEF5* function, coding DNA sequences for both the normal and risk variant *RAPGEF5* were cloned into a fluorescent expression vector and transfected into cultured canine kidney cells. The cellular location of the labeled RAPGEF5 proteins was then visually assessed. In contrast to the localization of the normal protein to the nucleus, the risk variant protein was located predominantly in the cytoplasm. With this altered location within the cell, the risk variant protein may alter the Wnt signaling pathway and contribute to the idiopathic epilepsy observed in Belgian Shepherds.

**Supplementary Information:**

The online version contains supplementary material available at 10.1186/s40575-024-00138-3.

## Introduction

Idiopathic epilepsy (IE) is an intractable and often debilitating neurological disorder frequently observed in dogs [1]. It is characterized by repeated seizure episodes that may be mild to severe with a typical age of onset between 2 and 4 years old [2]. Often, pharmacological intervention is necessary to control the seizures though some dogs are refractory to treatment and have a reduced quality of life. Thus, studies to understand the cause and etiology have targeted environmental and genetic inputs to reduce the incidence of canine IE. Efforts to control its incidence have focused on uncovering the genetic contributions to disease expression. Some dog breeds, such as the Belgian shepherd, are more likely to be affected by IE [2, 3] and thereby provide a useful population to study the genetic inputs. A previous genome association study using well-phenotyped Belgian shepherd dogs whose IE was defined based on the International Veterinary Task Force’s Tier 1 confidence level [4], revealed a four-base IE risk haplotype on canine chromosome (CFA) 14 [3]. The risk haplotype fell upstream of the *RAPGEF5* gene and upon further investigation, Belgian shepherd dogs having the risk haplotype were fully concordant with the presence of a disruptive 3-base pair (bp) insertion in exon 1 of the *RAPGEF5* gene [5].

The RAPGEF5 protein is involved in embryonic pattern formation through the Wnt signaling pathway [6]. The evolutionarily conserved Wnt family members are critically important in the establishment of cell polarity and in defining mitotic capacity and cell fate during embryogenesis [7]. Beyond its role in development, Wnt is also important in the physiological maintenance of tissues and perturbations in its signaling pathway can result in disease [8]. Upon activation, although there are two Wnt signaling pathways invoked [7], the most well-described is the canonical Wnt signaling mediated through a β-catenin dependent pathway and known to regulate proliferation [8] and neurodevelopment [9]. Signaling through the β‐catenin pathway involves translocation of β‐catenin to the nucleus whereupon it interacts with transcription factors to drive Wnt associated gene expression. Crucial in the signaling pathway is the nuclear translocation of β‐catenin [10] and in the absence of nuclear translocation, β‐catenin is degraded, impairing Wnt signaling [11].

Genetic studies of human epilepsy, including subjects diagnosed with IE, have uncovered associations with members of the Wnt pathway [12]. Additionally, a potential role for *RAPGEF5* in epilepsy has been implicated in humans [13] and in mouse models of epilepsy [14] suggesting a role for *RAPGEF5* in the development of canine IE. As mentioned above, a key modulator of the canonical Wnt pathway is β-catenin which must be translocated to the nucleus to effect its action, a process modulated by *RAPGEF5* [6]. The *RAPGEF5* 3-bp insertion found to be associated with IE in the Belgian shepherd introduces one additional alanine amino acid in the region adjacent to the conserved Dishevelled, Egl-10, and Pleckstrin (DEP) domain. The DEP domain plays a vital role in directing the RAPGEF5 protein to cellular membranes allowing its interactions with binding partners [15]. In silico analysis predicts the 3-bp insertion to be disruptive. We hypothesized that the presence of the *RAPGEF5* 3-bp insertion would disrupt the structure of the protein which in turn would result in an alteration of its cellular localization and thus implicating the *RAPGEF5* variant as a functional contributor to IE risk in the Belgian shepherd dog.

## Methods

The RAPGEF5 cDNA sequence was developed by aligning whole genome sequences (WGS) from 13 Belgian shepherds with CLC Genomics Workbench version 22.0.1 (Qiagen Digital Insights, Aarhus, Denmark). Whole blood samples from 13 Belgian Tervuren (n = 6 IE cases and n = 7 healthy controls over the age of 7 years old) were collected by the owner’s veterinarian and submitted to the study. These dogs share some ancestry where three dogs formed a trio of two parents and one IE case, two controls were half-siblings, two IE cases and two controls shared one common grandparent and one IE case and three controls were unrelated at the grandparent level. DNA was extracted from the blood samples with the QIAGEN QIAamp^®^ DNA Blood Mini Kit (QIAGEN Inc., Valencia, CA, USA) and quantified using a Qubit^®^ fluorometer (ThermoFisher Scientific, Waltham, MA, USA). DNA aliquots were stored at -20°C until submitted to Novogene (Novogene, Beijing, China) for WGS using the Illumina NovaSeq 6000 PE150 platform with an average of 12x coverage. Raw sequence data files were processed as previously described [16]. A consensus sequence of the cDNA was then aligned to the RAPGEF5 cDNA reference genome (CanFam3.1) for comparison. The SNPEff annotation program [17] characterizes the *RAPGEF5* 3-bp insertion as a disruptive inframe insertion with moderate effect. In silico analysis with the modeling program Phyre^2^ [18] predicts the 3-bp insertion to change the protein’s secondary structures (Additional File 1). The Belgian shepherd consensus sequence was used to synthesize the custom cDNA construct of the wildtype (WT) RAPGEF5 and formed the basis of a cDNA construct that included the 3 bp insertion IE risk variant (RISK) (GeneWiz, South Plainfield, NJ, USA). The synthesized sequences included the 5’ untranslated region (UTR) and 5’ Xhol and 3’ BamHI restriction enzyme sites (Additional File 2a). Sequences were verified and cloned into the expression vector mEGFP-N1 (Addgene, Watertown, MA, USA; Additional File 2b) to make a fusion protein with enhanced green fluorescent protein using standard practices [19]. The DNA quantity, quality, and expected fragment sizes for BamHI and Xhol restriction digest were verified (GeneWiz, South Plainfield, NJ, USA).

Since RAPGEF5 is expressed in kidney cells though not at high levels which might negatively influence the transfected cDNA expression [13], transient transfection of the cDNAs into the Madin-Darby Canine Kidney (MDCK) cells (ATCC CCL-34, Manassas, VA, USA), was done to ensure species-relevant expression. Cells were cultured in Eagle’s minimum essential medium (ATCC, Manassas, VA, USA) supplemented with 10% fetal bovine serum (MilliporeSigma, Burlington, MA, USA) and penicillin/streptomycin (Gibco, Grand Island, NY, USA) at 37^°^C in a humidified 5% CO_2_ incubator and plated the day prior to transfection at a density of 50,000 cells/well to achieve 70–80% confluency on the day of transfection. The expression vector plasmids were purified with the ZymoPure II Plasmid Midiprep Kit (Zymo Research, Irvine, CA, USA). The RAPGEF5 WT and RISK plasmid preps were verified by variant-specific PCR primers and Sanger sequencing as previously described [5]. Purified expression vector plasmid (2.5 µg) was transfected into cells using Lipofectamine 2000 according to the manufacturer’s instructions (3% v/v, Invitrogen, Carlsbad, CA, USA). To better resolve the localization of RAPGEF5-GFP fusion proteins to cellular structures, the nuclei of the cells were counterstained with Hoechst 33342, a blue fluorescence stain (Additional File 3 presents the composite image of GFP and Hoechst staining). The experimental unit included the plate wells and the transfections for each RAPGEF5 construct. Control groups included cells treated with pmaxGFP (Lonza, Basel, Switzerland) as the positive control for transfection, and non-treated cells without plasmid as the negative control. Cells from each control and treatment group were plated in triplicate across two plates in two separate experiments. To determine the RAPGEF5-GFP localization, live transfected cells were imaged using a Molecular Devices ImageXpress Micro Confocal fluorescence microscope (Molecular Devices, LLC., San Jose, CA, USA) at 40x magnification 17–23 h post-transfection and prior to cell division. Images were captured using laser channels for DAPI (blue fluorescence for nuclei which has a similar wavelength to Hoechst 33342) and FITC (green fluorescence of RAPGEF5-GFP fusion proteins). Fifteen images per treatment well were captured and fluorescent localization for RAPGEF5-GFP (cytoplasm, nucleus, or both) was quantified for every cell in each well. Nuclear localization was determined by the FITC fluorescence within the area encompassed by the blue fluorescence. Similarly, localization defined as cytoplasmic was determined by the FITC fluorescence outside of the blue labeled nuclei. Localization as both nuclear and cytoplasmic had FITC fluorescence observed covering the entire cell. Statistical significance of protein distribution, the percentage of total cells labeled, was determined by two-tailed t-tests for independent samples using the VassarStats web tool [20].

## Results

**Fig. 1:**
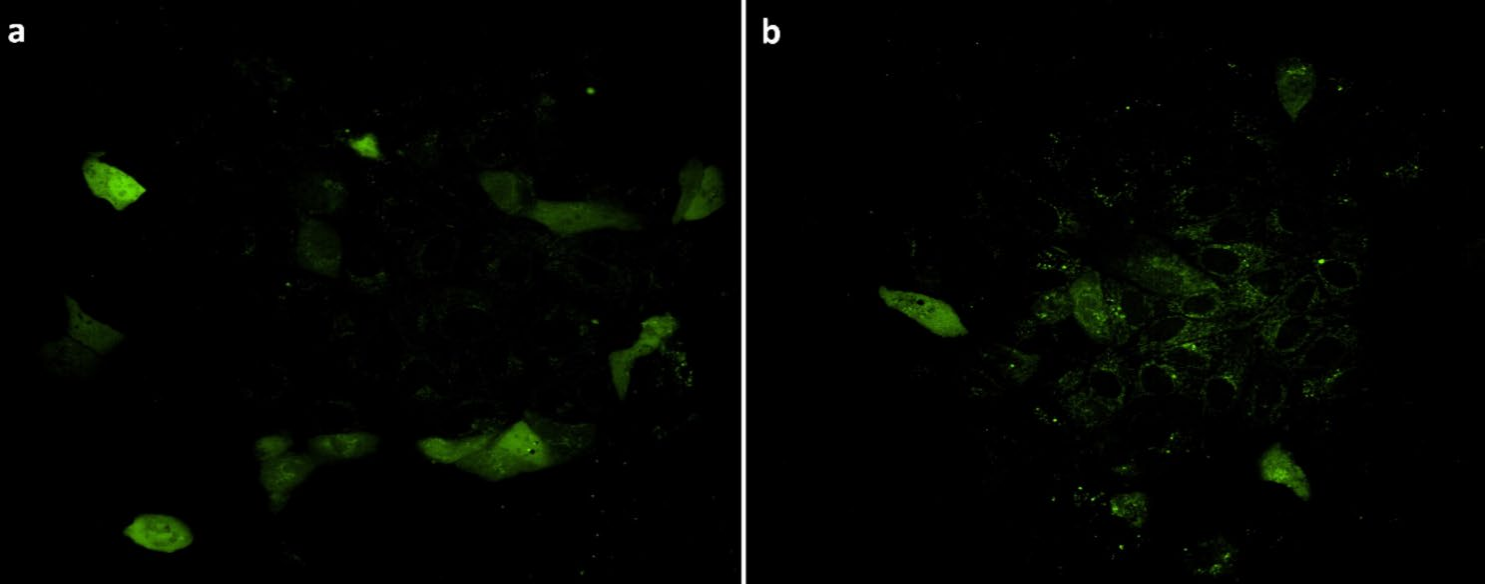
Green fluorescence of RAPGEF5-GFP fusion proteins in transfected MDCK cells for (**a**) wildtype and (**b**) risk variant that contains the 3-bp insertion

To determine whether the *RAPGEF5* 3-bp insertion risk variant functionally disrupted the protein and altered its cellular localization, cells were transfected with GFP-labeled wildtype and risk variant RAPGEF5 cDNAs. The cellular localization of the wildtype and risk RAPGEF5 variant proteins was quantified. Wildtype RAPGEF5 protein was found abundantly in the nucleus as well as in the cytoplasm. In contrast, the majority of the risk variant RAPGEF5 protein was localized just to the cytoplasm (Table 1; Fig. 1 and Additional File 3). The risk variant RAPGEF5 protein was detected in the cytoplasm of the transfected cells 1.5 times more than that observed for the wildtype protein (*p* < 0.05). Additionally, in a large proportion of cells transfected with the wildtype protein, both nuclear and cytoplasmic localization were observed and although not significant (*p* = 0.06), the combined localization of nucleus and cytoplasm in the same cell was 1.7 times more frequent than that seen for the risk variant.

**Table 1:** Cellular localization of RAPGEF5-GFP fusion proteins expressed from the RAPGEF5 wildtype and the idiopathic epilepsy risk variant, presented as a percentage of labeled cells (mean and standard error)

	Nuclei labeled (%)	Cytoplasm labeled (%)	Nuclei and Cytoplasm labeled (%)
Wildtype	6.21 + 2.31	45.12 + 6.14	48.67 + 7.46
Risk Variant	3.86 + 2.23	67.29 + 6.61	28.74 + 5.75
p-value	0.48	0.03	0.06

## Discussion

The relatively high prevalence of canine epilepsy [2] coupled with the knowledge that IE has a strong genetic component have been the impetus for genomic studies to identify contributing loci. One such risk locus, consisting of a 3-bp insertion in the *RAPGEF5* gene, was characterized for the Belgian shepherd dog and found in both related and unrelated dogs at a population frequency of 19.6%; additionally the average age of IE onset in dogs with the risk variant was 42 months of age [5]. In the present study, we demonstrated that the 3-bp insertion found to be associated with increased IE risk in the Belgian shepherd alters the cellular distribution of the RAPGEF5 protein. The data indicated that the wildtype RAPGEF5 was more frequently found in the nucleus and the nucleus and cytoplasm in combination, whereas the risk variant was significantly greater in the cytoplasmic compartment than that of the wildtype. *RAPGEF5* encodes a guanine nucleotide exchange factor involved in the highly conserved Wnt signaling pathway [6] that controls early embryonic pattern formation as well as having a prominent role in neurogenesis, neural differentiation, establishment of synapses, plasticity, and neural homeostasis [7, 21].

In the brain, the principal mediator of the Wnt brain signaling is the canonical β-catenin pathway which requires the translocation of β‐catenin to the nucleus where it then drives gene transcription [22]. Disruption of the Wnt/β‐catenin signaling pathway has been implicated in altered left-right patterning in the embryo [6], neurodegeneration [23] and both acute and chronic epilepsies [24]. While nuclear import is known to be a key modulatory step for β‐catenin’s action, the mechanism for nuclear translocation is less well-defined [25]. *RAPGEF5* has been shown to facilitate the translocation of β‐catenin to the nucleus. Specifically, a RAPGEF5 knockdown model showed decreased nuclear and increased cytoplasmic β‐catenin and reduced Wnt gene expression [6]. The proposed model [6] is that *RAPGEF5* maintains RAP proteins in the GTP-bound, activated state and the activated RAP proteins physically interact with β‐catenin promoting its translocation to the nucleus.

In the developing embryo, RAPGEF5 is localized in nuclei. The subcellular localization of wildtype RAPGEF5 is nuclear [6] consistent with a role of RAPGEF5 in β-catenin translocation. The RAPGEF5 protein with the risk 3-bp insertion was primarily found in the cytoplasm. In the absence of a fully functional RAPGEF5 protein, β‐catenin translocation would be impaired, and the embryonic pattern development and maintenance of adult tissues affected. This would suggest that the Wnt/β‐catenin signaling pathway may be negatively impacted in dogs with the risk variant, thereby demonstrating a functional role of the altered RAPGEF5 protein. Studies of epileptogenesis in the dog have identified anatomical disturbances, neurotransmission deficits, inflammation, and aberrations in networks all of which parallel human epilepsies [26]. A RAPGEF5 protein with reduced functionality could impair Wnt signaling due to reduced β‐catenin gene transcription. The effects of this could be manifested during development creating aberrations in the neural network circuitry and then through adulthood by a reduction in neurogenesis and maintenance. Importantly, because the variant did not abolish nuclear-associated RAPGEF5, some functionality of RAPGEF5 would be maintained. It is known that seizures have a bimodal effect promoting neuronal apoptosis and neurogenesis through the Wnt/β‐catenin pathway, presumably to moderate the damage induced by the seizure [24]. In humans, seizure episodes potentiate additional seizures and thus a defective RAPGEF5 protein could alter the normal development of the neuronal circuitry, and also impair seizure recovery resulting in greater seizure severity and/or frequency with increasing age. An iterative reduction in neurogenesis as a result of small, but accumulating effects of an impaired *RAPGEF5* might account for the 36-month median age of IE onset in the Belgian shepherd [3].

The 3-bp insertion associated with IE risk in the Belgian shepherd introduces one additional alanine into the *RAPGEF5* gene sequence of ten consecutive alanine amino acids. Homorepeats are often engaged in protein-protein interactions and subcellular trafficking of proteins [27]. They can be prone to expansion due to DNA strand slippage with a propensity to aggregate and lead to disease [27, 28]. Expanded homorepeats have been associated with neurodegenerative disease [27] and a modest alanine expansion in exon 1 of the poly (A) binding protein, nuclear 1 (PABPN1) gene is implicated in oculopharyngeal muscular dystrophy [29]. In humans and mice, the Aristaless Related Homeobox (ARX) gene has a polyalanine expansion mutation that is known to cause intellectual disability, epilepsy, and brain malformations [30] and has a similar spatial expression profile as RAPGEF5 determined by in situ hybridization [30]. Comparable to *RAPGEF5*, ARX positively regulates the Wnt/β-catenin signaling pathway [31]. Though the addition of one alanine in the RAPGEF5 protein is a small change, data in the present study shows that the introduction of a single alanine altered localization of RAPGEF5 protein within the cell.

The risk variant insertion is also adjacent to the DEP domain of RAPGEF5. The DEP domain is conserved across G-protein family members and coordinates spatial localization by directing the protein to cellular membranes [15] thereby permitting interaction with other binding partners. Thus, in addition to the expanded homorepeat, the insertion also has the potential to change the protein configuration of the DEP domain. This could also account for the observed altered localization to the cytoplasm in the risk variant form which in turn could impede intracellular signaling.

*RAPGEF5* has also been associated with neurological disorders. Fast ripples are an electrophysiological pattern preceding the onset of an epileptic episode and RAPGEF5 expression is downregulated in the hippocampal fast ripple region [14]. Magnetic resonance imaging of dogs during the peri-ictal phase of IE shows multiple regions of the brain involved to varying degrees with the hippocampus as a prominent node of engagement in network analyses [32]. The finding that *RAPGEF5* is associated with canine IE presents a new avenue of study. The altered cellular localization implies that the *RAPGEF5* locus identified by genomic association is a true risk locus for IE and further implicates Wnt/β-catenin in both human and canine epilepsy. A gene expression study of human infantile seizures revealed more than 70% of the genes associated with seizures were in the Wnt/β‐catenin signaling pathway [33]. Although β‐catenin has been a target of study in human epilepsies, its regulation has been less well-defined and it has not been considered for the dog. A parallel role between the two species offers potential interventions to mitigate IE in both.

The cellular location data reported here indicates that the variant identified in the Belgian shepherd likely reduces nuclear activity of β-catenin, but to what degree remains unclear. The impact of the presence of the variant in a given dog would be difficult to predict based upon an expected titration of β‐catenin signaling. Although GFP was a large protein added onto the N-terminus of the RAPGEF5 protein which could affect distribution in the transfection model, the observed distribution of the GFP-labeled wildtype protein being consistent with distribution in the literature [6] suggests that the GFP construct used is acceptable and the results reflect the true altered distribution of the variant form. Further studies using genetically modified mice carrying the *RAPGEF5* variant could yield additional insight into its role in epileptogenesis and provide a new animal model for epilepsy.

Given the complex nature of IE and the multifactorial inputs, identifying any and all risk factors contributing to IE in the dog and human is critically important to management, either through selective breeding in dogs, or treatment. The suggested role of *RAPGEF5* in modulating β-catenin gene transcription and its association with epilepsy in the dog offers insight into the development of IE in the human. Exploration of *RAPGEF5* genetic variants in human epilepsy could reveal risk and generalized mechanistic underpinnings of IE and give further evidence of a role for nuclear translocation of β‐catenin in epileptogenesis. A prevalent role of β‐catenin in the development of seizures suggests that therapeutic interventions targeting β‐catenin may be fruitful [24, 34]. Moderating nuclear translocation or β‐catenin overall concentrations through Wnt stimulatory molecules may be future treatment options.

## Electronic supplementary material

Below is the link to the electronic supplementary material.

**Additional file 1: Supplemental Fig. 1** Canine RAPGEF5 Predicted Secondary Structure Protein Models for WT (**a**) and RISK (**b**)

**Additional file 2: Supplemental Fig. 2a** RAPGEF5 cDNA in mEGFP-N1 Expression Vector Wild Type (WT) and Risk Variant (RISK) Sequence Alignment. Supplemental Fig. 2**b** mEGFP-N1 Plasmid Map

**Additional file 3: Supplemental Fig. 3** Confocal microscopic images of risk RAPGEF5-GFP fusion proteins in MDCK cells after transfection (40X magnification): (**a**) Overlay image of Hoechst 33342-stained nuclei and RAPGEF5-GFP fusion proteins; (**b**) RAPGEF5-GFP fusion proteins; (**c**) Hoechst 33342-stained nuclei

## Data Availability

No datasets were generated or analysed during the current study.
