# Supplementary material for: Cellular localization of a variant RAPGEF5 protein associated with idiopathic epilepsy risk in the Belgian shepherd
Source: Canine Med Genet. 2024 Sep 29;11:4. doi: 10.1186/s40575-024-00138-3 (PMC11439299; doi:10.1186/s40575-024-00138-3)

Additional file 1 Supplemental Fig. 1 Canine RAPGEF5 Predicted Secondary Structure Protein Models for WT (a) and RISK (b) with the circled area showing a change to the alpha helix structure (magenta color). Models created in Phyre^2 with JSmol (Kelley, L., Mezulis, S., Yates, C. et al. The Phyre2 web portal for protein modeling, prediction and analysis. Nat Protoc 10, 845–858, 2015; http://www.jmol.org/).

a.

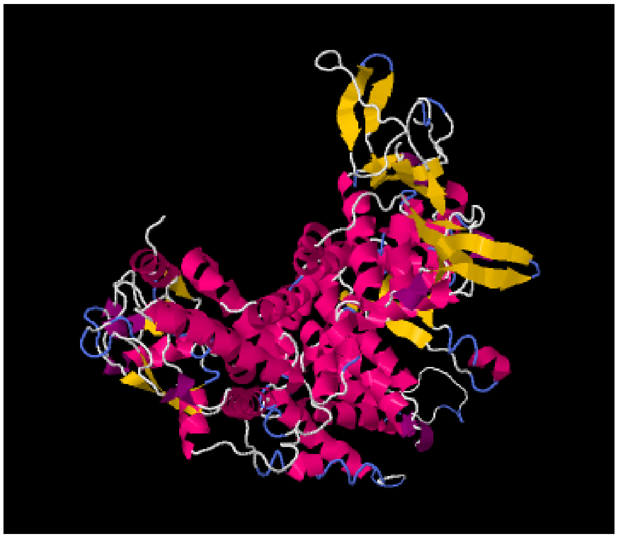

b.

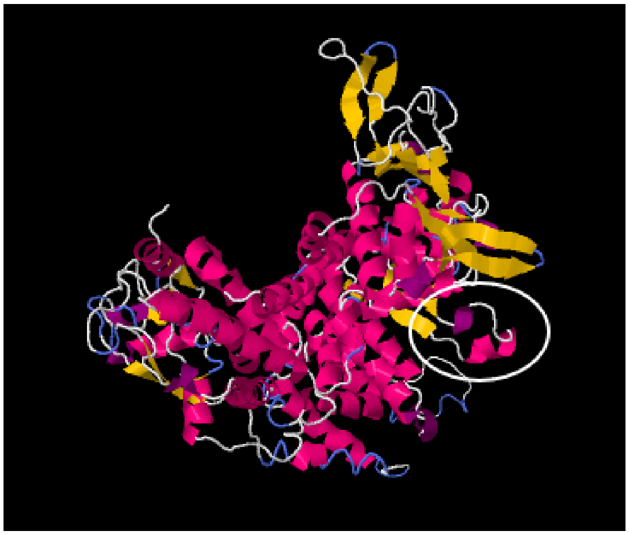

Supplement: Supplementary file 1 — Additional file 1: Supplemental Fig. 1 Canine RAPGEF5 Predicted Secondary Structure Protein Models for WT (a) and RISK (b) [file 40575_2024_138_MOESM1_ESM.pdf]
