# Supplementary material for: Cellular localization of a variant RAPGEF5 protein associated with idiopathic epilepsy risk in the Belgian shepherd
Source: Canine Med Genet. 2024 Sep 29;11:4. doi: 10.1186/s40575-024-00138-3 (PMC11439299; doi:10.1186/s40575-024-00138-3)

Additional file 2 Supplemental Fig. 2a

RAPGEF5 cDNA in mEGFP-N1 Expression Vector Wild Type (WT) and Risk Variant (RISK) Sequence Alignment (CLC Genomics Workbench 22.0.1). Location of restriction enzymes, 5' UTR, gene start/end, and INDEL indicated by an asterik (*).

```
                                      20         40         60
RAPGEF5_WT_ mEGFP-N1    CGTTACATAA CTTACGGTAA ATGGCCCGCC TGGCTGACCG CCCAACGACC CCCGCCCATT 60
RAPGEF5_RISK_mEGFP-N1   CGTTACATAA CTTACGGTAA ATGGCCCGCC TGGCTGACCG CCCAACGACC CCCGCCCATT 60
Consensus               CGTTACATAA CTTACGGTAA ATGGCCCGCC TGGCTGACCG CCCAACGACC CCCGCCCATT
Conservation            100% / 0%

                                      80         100        120
RAPGEF5_WT_ mEGFP-N1    GACGTCAATA ATGACGTATG TTCCCATAGT AACGCCAATA GGGACTTTCC ATTGACGTCA 120
RAPGEF5_RISK_mEGFP-N1   GACGTCAATA ATGACGTATG TTCCCATAGT AACGCCAATA GGGACTTTCC ATTGACGTCA 120
Consensus               GACGTCAATA ATGACGTATG TTCCCATAGT AACGCCAATA GGGACTTTCC ATTGACGTCA
Conservation            100% / 0%

                                      140        160        180
RAPGEF5_WT_ mEGFP-N1    ATGGGTGGAG TATTTACGGT AAACTGCCCA CTTGGCAGTA CATCAAGTGT ATCATATGCC 180
RAPGEF5_RISK_mEGFP-N1   ATGGGTGGAG TATTTACGGT AAACTGCCCA CTTGGCAGTA CATCAAGTGT ATCATATGCC 180
Consensus               ATGGGTGGAG TATTTACGGT AAACTGCCCA CTTGGCAGTA CATCAAGTGT ATCATATGCC
Conservation            100% / 0%

                                      200        220        240
RAPGEF5_WT_ mEGFP-N1    AAGTACGCCC CCTATTGACG TCAATGACGG TAAATGGCCC GCCTGGCATT ATGCCCAGTA 240
RAPGEF5_RISK_mEGFP-N1   AAGTACGCCC CCTATTGACG TCAATGACGG TAAATGGCCC GCCTGGCATT ATGCCCAGTA 240
Consensus               AAGTACGCCC CCTATTGACG TCAATGACGG TAAATGGCCC GCCTGGCATT ATGCCCAGTA
Conservation            100% / 0%

                                      260        280        300
RAPGEF5_WT_ mEGFP-N1    CATGACCTTA TGGGACTTTC CTACTTGGCA GTACATCTAC GTATTAGTCA TCGCTATTAC 300
RAPGEF5_RISK_mEGFP-N1   CATGACCTTA TGGGACTTTC CTACTTGGCA GTACATCTAC GTATTAGTCA TCGCTATTAC 300
Consensus               CATGACCTTA TGGGACTTTC CTACTTGGCA GTACATCTAC GTATTAGTCA TCGCTATTAC
Conservation            100% / 0%

                                      320        340        360
RAPGEF5_WT_ mEGFP-N1    CATGGTGATG CGGTTTTGGC AGTACATCAA TGGGCGTGGA TAGCGGTTTG ACTCACGGGG 360
RAPGEF5_RISK_mEGFP-N1   CATGGTGATG CGGTTTTGGC AGTACATCAA TGGGCGTGGA TAGCGGTTTG ACTCACGGGG 360
Consensus               CATGGTGATG CGGTTTTGGC AGTACATCAA TGGGCGTGGA TAGCGGTTTG ACTCACGGGG
Conservation            100% / 0%

                                      380        400        420
RAPGEF5_WT_ mEGFP-N1    ATTTCCAAGT CTCCACCCCA TTGACGTCAA TGGGAGTTTG TTTTGGCACC AAAATCAACG 420
RAPGEF5_RISK_mEGFP-N1   ATTTCCAAGT CTCCACCCCA TTGACGTCAA TGGGAGTTTG TTTTGGCACC AAAATCAACG 420
Consensus               ATTTCCAAGT CTCCACCCCA TTGACGTCAA TGGGAGTTTG TTTTGGCACC AAAATCAACG
Conservation            100% / 0%

                                      440        460        480
RAPGEF5_WT_ mEGFP-N1    GGACTTTCCA AAATGTCGTA ACAACTCCGC CCCATTGACG CAAATGGGCG GTAGGCGTGT 480
RAPGEF5_RISK_mEGFP-N1   GGACTTTCCA AAATGTCGTA ACAACTCCGC CCCATTGACG CAAATGGGCG GTAGGCGTGT 480
Consensus               GGACTTTCCA AAATGTCGTA ACAACTCCGC CCCATTGACG CAAATGGGCG GTAGGCGTGT
Conservation            100% / 0%

                                      500        520        540
RAPGEF5_WT_ mEGFP-N1    ACGGTGGGAG GTCTATATAA GCAGAGCTGG TTTAGTGAAC CGTCAGATCC GCTAGCGCTA 540
RAPGEF5_RISK_mEGFP-N1   ACGGTGGGAG GTCTATATAA GCAGAGCTGG TTTAGTGAAC CGTCAGATCC GCTAGCGCTA 540
Consensus               ACGGTGGGAG GTCTATATAA GCAGAGCTGG TTTAGTGAAC CGTCAGATCC GCTAGCGCTA
Conservation            100% / 0%

                              XhoI   *560        580        600
RAPGEF5_WT_ mEGFP-N1    CCGGACTCAG ATCTCGAGTG AATTATCTGT TTGGTTCGGG GCCCGGCGCA CCTGGGCGGG 600   RAPGEF5 gene 5' UTR
RAPGEF5_RISK_mEGFP-N1   CCGGACTCAG ATCTCGAGTG AATTATCTGT TTGGTTCGGG GCCCGGCGCA CCTGGGCGGG 600
Consensus               CCGGACTCAG ATCTCGAGTG AATTATCTGT TTGGTTCGGG GCCCGGCGCA CCTGGGCGGG
Conservation            100% / 0%

                                      620        640        660
RAPGEF5_WT_ mEGFP-N1    GAGGGGGAGG CGCGCGGGGC CGAGCCTGTC GGGGGCCGCG GCGCGCGCCA ATGGGCAGCG 660
RAPGEF5_RISK_mEGFP-N1   GAGGGGGAGG CGCGCGGGGC CGAGCCTGTC GGGGGCCGCG GCGCGCGCCA ATGGGCAGCG 660
Consensus               GAGGGGGAGG CGCGCGGGGC CGAGCCTGTC GGGGGCCGCG GCGCGCGCCA ATGGGCAGCG
Conservation            100% / 0%

                                      680        700        720
RAPGEF5_WT_ mEGFP-N1    GCTGGCAGCG CGCCCCGCGC CCGAGCCGCA GTCGCGCCCT CCCGCTGCCT GGCGAAGGCG 720
RAPGEF5_RISK_mEGFP-N1   GCTGGCAGCG CGCCCCGCGC CCGAGCCGCA GTCGCGCCCT CCCGCTGCCT GGCGAAGGCG 720
Consensus               GCTGGCAGCG CGCCCCGCGC CCGAGCCGCA GTCGCGCCCT CCCGCTGCCT GGCGAAGGCG
Conservation            100% / 0%
```

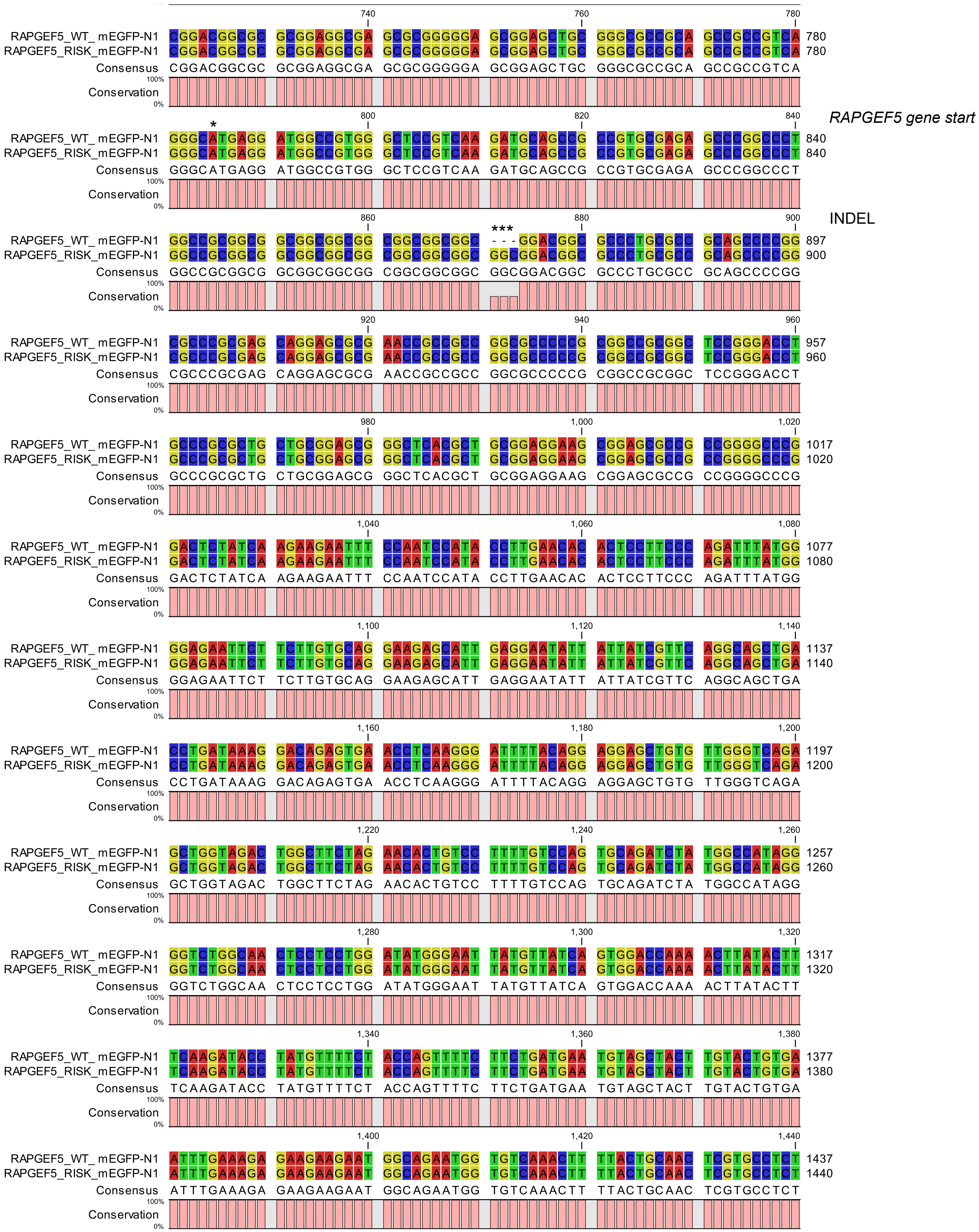
740 760 780
RAPGEF5_WT_ mEGFP-N1 CGGACGGCGC GCGGAGGCGA GCGCGGGGGA GCGGAGCTGC GGGCGCCGCA GCCGCCGTCA 780
RAPGEF5_RISK_mEGFP-N1 CGGACGGCGC GCGGAGGCGA GCGCGGGGGA GCGGAGCTGC GGGCGCCGCA GCCGCCGTCA 780
Consensus CGGACGGCGC GCGGAGGCGA GCGCGGGGGA GCGGAGCTGC GGGCGCCGCA GCCGCCGTCA
Conservation
*
800 820 840
RAPGEF5 gene start
RAPGEF5_WT_ mEGFP-N1 GGGCATGAGG ATGGCCGTGG GCTCCGTCAA GATGCAGCCG CCGTGCGAGA GCCCGGCCCT 840
RAPGEF5_RISK_mEGFP-N1 GGGCATGAGG ATGGCCGTGG GCTCCGTCAA GATGCAGCCG CCGTGCGAGA GCCCGGCCCT 840
Consensus GGGCATGAGG ATGGCCGTGG GCTCCGTCAA GATGCAGCCG CCGTGCGAGA GCCCGGCCCT
Conservation
***
860 880 900
INDEL
RAPGEF5_WT_ mEGFP-N1 GGCCGCGGCG GCGGCGGCGG CGGCGGCGGC ---GGACGGC GCCCTGCGCC GCAGCCCCGG 897
RAPGEF5_RISK_mEGFP-N1 GGCCGCGGCG GCGGCGGCGG CGGCGGCGGC GGCGGACGGC GCCCTGCGCC GCAGCCCCGG 900
Consensus GGCCGCGGCG GCGGCGGCGG CGGCGGCGGC GGCGGACGGC GCCCTGCGCC GCAGCCCCGG
Conservation
920 940 960
RAPGEF5_WT_ mEGFP-N1 CGCCCGCGAG CAGGAGCGCG AACCGCCGCC GGCGCCCCCG CGGCCGCGGC TCCGGGACCT 957
RAPGEF5_RISK_mEGFP-N1 CGCCCGCGAG CAGGAGCGCG AACCGCCGCC GGCGCCCCCG CGGCCGCGGC TCCGGGACCT 960
Consensus CGCCCGCGAG CAGGAGCGCG AACCGCCGCC GGCGCCCCCG CGGCCGCGGC TCCGGGACCT
Conservation
980 1,000 1,020
RAPGEF5_WT_ mEGFP-N1 GCCCGCGCTG CTGCGGAGCG GGCTCACGCT GCGGAGGAAG CGGAGCGCCG CCGGGGCCCG 1017
RAPGEF5_RISK_mEGFP-N1 GCCCGCGCTG CTGCGGAGCG GGCTCACGCT GCGGAGGAAG CGGAGCGCCG CCGGGGCCCG 1020
Consensus GCCCGCGCTG CTGCGGAGCG GGCTCACGCT GCGGAGGAAG CGGAGCGCCG CCGGGGCCCG
Conservation
1,040 1,060 1,080
RAPGEF5_WT_ mEGFP-N1 GACTCTATCA AGAAGAATTT CCAATCCATA CCTTGAACAC ACTCCTTCCC AGATTTATGG 1077
RAPGEF5_RISK_mEGFP-N1 GACTCTATCA AGAAGAATTT CCAATCCATA CCTTGAACAC ACTCCTTCCC AGATTTATGG 1080
Consensus GACTCTATCA AGAAGAATTT CCAATCCATA CCTTGAACAC ACTCCTTCCC AGATTTATGG
Conservation
1,100 1,120 1,140
RAPGEF5_WT_ mEGFP-N1 GGAGAATTCT TCTTGTGCAG GAAGAGCATT GAGGAATATT ATTATCGTTC AGGCAGCTGA 1137
RAPGEF5_RISK_mEGFP-N1 GGAGAATTCT TCTTGTGCAG GAAGAGCATT GAGGAATATT ATTATCGTTC AGGCAGCTGA 1140
Consensus GGAGAATTCT TCTTGTGCAG GAAGAGCATT GAGGAATATT ATTATCGTTC AGGCAGCTGA
Conservation
1,160 1,180 1,200
RAPGEF5_WT_ mEGFP-N1 CCTGATAAAG GACAGAGTGA ACCTCAAGGG ATTTTACAGG AGGAGCTGTG TTGGGTCAGA 1197
RAPGEF5_RISK_mEGFP-N1 CCTGATAAAG GACAGAGTGA ACCTCAAGGG ATTTTACAGG AGGAGCTGTG TTGGGTCAGA 1200
Consensus CCTGATAAAG GACAGAGTGA ACCTCAAGGG ATTTTACAGG AGGAGCTGTG TTGGGTCAGA
Conservation
1,220 1,240 1,260
RAPGEF5_WT_ mEGFP-N1 GCTGGTAGAC TGGCTTCTAG AACACTGTCC TTTTGTCCAG TGCAGATCTA TGGCCATAGG 1257
RAPGEF5_RISK_mEGFP-N1 GCTGGTAGAC TGGCTTCTAG AACACTGTCC TTTTGTCCAG TGCAGATCTA TGGCCATAGG 1260
Consensus GCTGGTAGAC TGGCTTCTAG AACACTGTCC TTTTGTCCAG TGCAGATCTA TGGCCATAGG
Conservation
1,280 1,300 1,320
RAPGEF5_WT_ mEGFP-N1 GGTCTGGCAA CTCCTCCTGG ATATGGGAAT TATGTTATCA GTGGACCAAA ACTTATACTT 1317
RAPGEF5_RISK_mEGFP-N1 GGTCTGGCAA CTCCTCCTGG ATATGGGAAT TATGTTATCA GTGGACCAAA ACTTATACTT 1320
Consensus GGTCTGGCAA CTCCTCCTGG ATATGGGAAT TATGTTATCA GTGGACCAAA ACTTATACTT
Conservation
1,340 1,360 1,380
RAPGEF5_WT_ mEGFP-N1 TCAAGATACC TATGTTTTCT ACCAGTTTTC TTCTGATGAA TGTAGCTACT TGTACTGTGA 1377
RAPGEF5_RISK_mEGFP-N1 TCAAGATACC TATGTTTTCT ACCAGTTTTC TTCTGATGAA TGTAGCTACT TGTACTGTGA 1380
Consensus TCAAGATACC TATGTTTTCT ACCAGTTTTC TTCTGATGAA TGTAGCTACT TGTACTGTGA
Conservation
1,400 1,420 1,440
RAPGEF5_WT_ mEGFP-N1 ATTTGAAAGA GAAGAAGAAT GGCAGAATGG TGTCAAACTT TTACTGCAAC TCGTGCCTCT 1437
RAPGEF5_RISK_mEGFP-N1 ATTTGAAAGA GAAGAAGAAT GGCAGAATGG TGTCAAACTT TTACTGCAAC TCGTGCCTCT 1440
Consensus ATTTGAAAGA GAAGAAGAAT GGCAGAATGG TGTCAAACTT TTACTGCAAC TCGTGCCTCT
Conservation

```
                                    1,460                  1,480                  1,500
RAPGEF5_WT_ mEGFP-N1   CATTCCTGCT AGAGCTGGCA TCTGTGAACT GTCTCATCAG AAAATCGAAG ACTCGGAAGA 1497
RAPGEF5_RISK_mEGFP-N1  CATTCCTGCT AGAGCTGGCA TCTGTGAACT GTCTCATCAG AAAATCGAAG ACTCGGAAGA 1500
Consensus              CATTCCTGCT AGAGCTGGCA TCTGTGAACT GTCTCATCAG AAAATCGAAG ACTCGGAAGA
Conservation (100% / 0%)

                                    1,520                  1,540                  1,560
RAPGEF5_WT_ mEGFP-N1   AAGCAGTGAT GAAATTCTTG CTCGCCTAAC ATCTGCGGTG CAGAGAGAAC TAGCAGCTGT 1557
RAPGEF5_RISK_mEGFP-N1  AAGCAGTGAT GAAATTCTTG CTCGCCTAAC ATCTGCGGTG CAGAGAGAAC TAGCAGCTGT 1560
Consensus              AAGCAGTGAT GAAATTCTTG CTCGCCTAAC ATCTGCGGTG CAGAGAGAAC TAGCAGCTGT
Conservation (100% / 0%)

                                    1,580                  1,600                  1,620
RAPGEF5_WT_ mEGFP-N1   TATTGCTTTG AAAGCAAGGA AGTCTGCAAT TGAACAAGAT GAAGAAAACA GTGACAAACG 1617
RAPGEF5_RISK_mEGFP-N1  TATTGCTTTG AAAGCAAGGA AGTCTGCAAT TGAACAAGAT GAAGAAAACA GTGACAAACG 1620
Consensus              TATTGCTTTG AAAGCAAGGA AGTCTGCAAT TGAACAAGAT GAAGAAAACA GTGACAAACG
Conservation (100% / 0%)

                                    1,640                  1,660                  1,680
RAPGEF5_WT_ mEGFP-N1   CGTAATTGTG TCAGAAGCTG GAGGAGTTCC AGATTCTCAG GCGGGAGTGA TGTGCAAACT 1677
RAPGEF5_RISK_mEGFP-N1  CGTAATTGTG TCAGAAGCTG GAGGAGTTCC AGATTCTCAG GCGGGAGTGA TGTGCAAACT 1680
Consensus              CGTAATTGTG TCAGAAGCTG GAGGAGTTCC AGATTCTCAG GCGGGAGTGA TGTGCAAACT
Conservation (100% / 0%)

                                    1,700                  1,720                  1,740
RAPGEF5_WT_ mEGFP-N1   GCAGGAGAGA GATGACATTG GACGAATCGA ACTGGTCCAG AAGCTGGCAA GAGAAAACTG 1737
RAPGEF5_RISK_mEGFP-N1  GCAGGAGAGA GATGACATTG GACGAATCGA ACTGGTCCAG AAGCTGGCAA GAGAAAACTG 1740
Consensus              GCAGGAGAGA GATGACATTG GACGAATCGA ACTGGTCCAG AAGCTGGCAA GAGAAAACTG
Conservation (100% / 0%)

                                    1,760                  1,780                  1,800
RAPGEF5_WT_ mEGFP-N1   TCAGTTTCTA CAGGCAGACA AAAAAGAACA GGAGAAATCT GAACACGATG ATGAAGTGGC 1797
RAPGEF5_RISK_mEGFP-N1  TCAGTTTCTA CAGGCAGACA AAAAAGAACA GGAGAAATCT GAACACGATG ATGAAGTGGC 1800
Consensus              TCAGTTTCTA CAGGCAGACA AAAAAGAACA GGAGAAATCT GAACACGATG ATGAAGTGGC
Conservation (100% / 0%)

                                    1,820                  1,840                  1,860
RAPGEF5_WT_ mEGFP-N1   GACCGTTCAG GTTACGGAGC AGGAGCGGAA CGTCCTGGTG CTGAAGAAAG TGCAGGGCTG 1857
RAPGEF5_RISK_mEGFP-N1  GACCGTTCAG GTTACGGAGC AGGAGCGGAA CGTCCTGGTG CTGAAGAAAG TGCAGGGCTG 1860
Consensus              GACCGTTCAG GTTACGGAGC AGGAGCGGAA CGTCCTGGTG CTGAAGAAAG TGCAGGGCTG
Conservation (100% / 0%)

                                    1,880                  1,900                  1,920
RAPGEF5_WT_ mEGFP-N1   TGGCCCGGCC CCCCCGGCCG GCAGCGCCGA GAGTGATTGG AGATACGTGG TCGTGTCCGG 1917
RAPGEF5_RISK_mEGFP-N1  TGGCCCGGCC CCCCCGGCCG GCAGCGCCGA GAGTGATTGG AGATACGTGG TCGTGTCCGG 1920
Consensus              TGGCCCGGCC CCCCCGGCCG GCAGCGCCGA GAGTGATTGG AGATACGTGG TCGTGTCCGG
Conservation (100% / 0%)

                                    1,940                  1,960                  1,980
RAPGEF5_WT_ mEGFP-N1   GACCCCGGAG AAGATTTTGG AGCACCTTTT GAATGACTTG CACCTGGAAG AAGTCCAGGA 1977
RAPGEF5_RISK_mEGFP-N1  GACCCCGGAG AAGATTTTGG AGCACCTTTT GAATGACTTG CACCTGGAAG AAGTCCAGGA 1980
Consensus              GACCCCGGAG AAGATTTTGG AGCACCTTTT GAATGACTTG CACCTGGAAG AAGTCCAGGA
Conservation (100% / 0%)

                                    2,000                  2,020                  2,040
RAPGEF5_WT_ mEGFP-N1   CAAAGAAACA GAGACCCTCC TTGATGACTT CCTTCTCACG TACACTGTCT TCATGACAAC 2037
RAPGEF5_RISK_mEGFP-N1  CAAAGAAACA GAGACCCTCC TTGATGACTT CCTTCTCACG TACACTGTCT TCATGACAAC 2040
Consensus              CAAAGAAACA GAGACCCTCC TTGATGACTT CCTTCTCACG TACACTGTCT TCATGACAAC
Conservation (100% / 0%)

                                    2,060                  2,080                  2,100
RAPGEF5_WT_ mEGFP-N1   TGATGACTTG TGCCAGGCGC TGTTGAGGCA CTATTCTGCT AAGAAGTATC AAGGCAAAGA 2097
RAPGEF5_RISK_mEGFP-N1  TGATGACTTG TGCCAGGCGC TGTTGAGGCA CTATTCTGCT AAGAAGTATC AAGGCAAAGA 2100
Consensus              TGATGACTTG TGCCAGGCGC TGTTGAGGCA CTATTCTGCT AAGAAGTATC AAGGCAAAGA
Conservation (100% / 0%)

                                    2,120                  2,140                  2,160
RAPGEF5_WT_ mEGFP-N1   AGAAAACTCA GATGTTCCTT GTCGGAAACG TAAGGTCTTA CATCTTGTGT CCCAATGGAT 2157
RAPGEF5_RISK_mEGFP-N1  AGAAAACTCA GATGTTCCTT GTCGGAAACG TAAGGTCTTA CATCTTGTGT CCCAATGGAT 2160
Consensus              AGAAAACTCA GATGTTCCTT GTCGGAAACG TAAGGTCTTA CATCTTGTGT CCCAATGGAT
Conservation (100% / 0%)
```

```
                              2,180                 2,200                 2,220
RAPGEF5_WT_ mEGFP-N1    TGCTCTCTAC AAAGACTGGT TACATGAAGA TGAACATTCA AAAATGTTCT TAAAGACCAT 2217
RAPGEF5_RISK_mEGFP-N1   TGCTCTCTAC AAAGACTGGT TACATGAAGA TGAACATTCA AAAATGTTCT TAAAGACCAT 2220
Consensus               TGCTCTCTAC AAAGACTGGT TACATGAAGA TGAACATTCA AAAATGTTCT TAAAGACCAT
100%
Conservation
0%
                              2,240                 2,260                 2,280
RAPGEF5_WT_ mEGFP-N1    ATACAGGAAT GTACTGGATG ATGTATATGA ATATCCAATA CTTGAAAAAG AATTGAAAGA 2277
RAPGEF5_RISK_mEGFP-N1   ATACAGGAAT GTACTGGATG ATGTATATGA ATATCCAATA CTTGAAAAAG AATTGAAAGA 2280
Consensus               ATACAGGAAT GTACTGGATG ATGTATATGA ATATCCAATA CTTGAAAAAG AATTGAAAGA
100%
Conservation
0%
                              2,300                 2,320                 2,340
RAPGEF5_WT_ mEGFP-N1    ATTTCAAAAG ATACTTGGAA TGCACCGCCG TCACACCGTA GATGAATATT CACCACAAAG 2337
RAPGEF5_RISK_mEGFP-N1   ATTTCAAAAG ATACTTGGAA TGCACCGCCG TCACACCGTA GATGAATATT CACCACAAAG 2340
Consensus               ATTTCAAAAG ATACTTGGAA TGCACCGCCG TCACACCGTA GATGAATATT CACCACAAAG
100%
Conservation
0%
                              2,360                 2,380                 2,400
RAPGEF5_WT_ mEGFP-N1    GAAGAATAAA GCCCTTTTCC ACCAATTCAG TCTCAAGGAG AACTGGCTCC AGCACAGAGG 2397
RAPGEF5_RISK_mEGFP-N1   GAAGAATAAA GCCCTTTTCC ACCAATTCAG TCTCAAGGAG AACTGGCTCC AGCACAGAGG 2400
Consensus               GAAGAATAAA GCCCTTTTCC ACCAATTCAG TCTCAAGGAG AACTGGCTCC AGCACAGAGG
100%
Conservation
0%
                              2,420                 2,440                 2,460
RAPGEF5_WT_ mEGFP-N1    AACTGTGACT GAAACAGAGG AAATTTTTTG CCACGTGTAT ATAACGGAGC ACTCCTATGT 2457
RAPGEF5_RISK_mEGFP-N1   AACTGTGACT GAAACAGAGG AAATTTTTTG CCACGTGTAT ATAACGGAGC ACTCCTATGT 2460
Consensus               AACTGTGACT GAAACAGAGG AAATTTTTTG CCACGTGTAT ATAACGGAGC ACTCCTATGT
100%
Conservation
0%
                              2,480                 2,500                 2,520
RAPGEF5_WT_ mEGFP-N1    CAGCGTGAAG GCAAAAGTTT CCAGTACAGC ACATGAGATC CTGAAAGTTG TGGCAGAAAA 2517
RAPGEF5_RISK_mEGFP-N1   CAGCGTGAAG GCAAAAGTTT CCAGTACAGC ACATGAGATC CTGAAAGTTG TGGCAGAAAA 2520
Consensus               CAGCGTGAAG GCAAAAGTTT CCAGTACAGC ACATGAGATC CTGAAAGTTG TGGCAGAAAA
100%
Conservation
0%
                              2,540                 2,560                 2,580
RAPGEF5_WT_ mEGFP-N1    GATCCAGCAT GCAGAAGAGG ATCTGGCTCT GGTGGCCGTC ACATTCTCTG GGGAAAAGCA 2577
RAPGEF5_RISK_mEGFP-N1   GATCCAGCAT GCAGAAGAGG ATCTGGCTCT GGTGGCCGTC ACATTCTCTG GGGAAAAGCA 2580
Consensus               GATCCAGCAT GCAGAAGAGG ATCTGGCTCT GGTGGCCGTC ACATTCTCTG GGGAAAAGCA
100%
Conservation
0%
                              2,600                 2,620                 2,640
RAPGEF5_WT_ mEGFP-N1    TGAACTTCAG CCAAATGACT TGGCCATCTC CAAATCGCTT GAAGCATCTG GTCGGATATA 2637
RAPGEF5_RISK_mEGFP-N1   TGAACTTCAG CCAAATGACT TGGCCATCTC CAAATCGCTT GAAGCATCTG GTCGGATATA 2640
Consensus               TGAACTTCAG CCAAATGACT TGGCCATCTC CAAATCGCTT GAAGCATCTG GTCGGATATA
100%
Conservation
0%
                              2,660                 2,680                 2,700
RAPGEF5_WT_ mEGFP-N1    TGTCTACCGA AAAGACCTAG CCGATACTTT GAACCCATTT GCAGAAAATG AGGACTCGCA 2697
RAPGEF5_RISK_mEGFP-N1   TGTCTACCGA AAAGACCTAG CCGATACTTT GAACCCATTT GCAGAAAATG AGGACTCGCA 2700
Consensus               TGTCTACCGA AAAGACCTAG CCGATACTTT GAACCCATTT GCAGAAAATG AGGACTCGCA
100%
Conservation
0%
                              2,720                 2,740                 2,760
RAPGEF5_WT_ mEGFP-N1    GCAAAGGTCG ATGAGGATTT TGGGAATGAA CACTTGGGAT CTTGCTCTGG AATTAATGAA 2757
RAPGEF5_RISK_mEGFP-N1   GCAAAGGTCG ATGAGGATTT TGGGAATGAA CACTTGGGAT CTTGCTCTGG AATTAATGAA 2760
Consensus               GCAAAGGTCG ATGAGGATTT TGGGAATGAA CACTTGGGAT CTTGCTCTGG AATTAATGAA
100%
Conservation
0%
                              2,780                 2,800                 2,820
RAPGEF5_WT_ mEGFP-N1    TTTTGATTGG AGTCTCTTCA ATTCAATTCA CGAGCAAGAG CTGATCTACT TTACCTTCAG 2817
RAPGEF5_RISK_mEGFP-N1   TTTTGATTGG AGTCTCTTCA ATTCAATTCA CGAGCAAGAG CTGATCTACT TTACCTTCAG 2820
Consensus               TTTTGATTGG AGTCTCTTCA ATTCAATTCA CGAGCAAGAG CTGATCTACT TTACCTTCAG
100%
Conservation
0%
                              2,840                 2,860                 2,880
RAPGEF5_WT_ mEGFP-N1    CCGACAAGGC AGTGGAGAGC ACACGGTGAA CCTCAGCCTT CTGCTGCAGA GATGCAATGA 2877
RAPGEF5_RISK_mEGFP-N1   CCGACAAGGC AGTGGAGAGC ACACGGTGAA CCTCAGCCTT CTGCTGCAGA GATGCAATGA 2880
Consensus               CCGACAAGGC AGTGGAGAGC ACACGGTGAA CCTCAGCCTT CTGCTGCAGA GATGCAATGA
100%
Conservation
0%
```

```
                                        2,900                          2,920                          2,940
RAPGEF5_WT_ mEGFP-N1    AGTCCAGCTC TGGGTGGCCA CGGAGATCCT GCTCTGCAGC CAGCTGGGCA AGCGAGTGCA 2937
RAPGEF5_RISK_mEGFP-N1   AGTCCAGCTC TGGGTGGCCA CGGAGATCCT GCTCTGCAGC CAGCTGGGCA AGCGAGTGCA 2940
            Consensus   AGTCCAGCTC TGGGTGGCCA CGGAGATCCT GCTCTGCAGC CAGCTGGGCA AGCGAGTGCA
    100%
Conservation
    0%
                                        2,960                          2,980                          3,000
RAPGEF5_WT_ mEGFP-N1    GCTGGTGAAA AAATTCATCA AAATCGCTGC GCACTGCAAA GCCCAGAGAA ACCTGAATTC 2997
RAPGEF5_RISK_mEGFP-N1   GCTGGTGAAA AAATTCATCA AAATCGCTGC GCACTGCAAA GCCCAGAGAA ACCTGAATTC 3000
            Consensus   GCTGGTGAAA AAATTCATCA AAATCGCTGC GCACTGCAAA GCCCAGAGAA ACCTGAATTC
    100%
Conservation
    0%
                                        3,020                          3,040                          3,060
RAPGEF5_WT_ mEGFP-N1    CTTCTTCGCT ATTGTGATGG GTCTCAACAC TGCTTCTGTC AGCCGGCTGT CACAGACCTG 3057
RAPGEF5_RISK_mEGFP-N1   CTTCTTCGCT ATTGTGATGG GTCTCAACAC TGCTTCTGTC AGCCGGCTGT CACAGACCTG 3060
            Consensus   CTTCTTCGCT ATTGTGATGG GTCTCAACAC TGCTTCTGTC AGCCGGCTGT CACAGACCTG
    100%
Conservation
    0%
                                        3,080                          3,100                          3,120
RAPGEF5_WT_ mEGFP-N1    GGAGAAAATC CCTGGGAAGT TTAAGAAACT TTTCTCTGAA CTTGAAAGCT TAACAGATCC 3117
RAPGEF5_RISK_mEGFP-N1   GGAGAAAATC CCTGGGAAGT TTAAGAAACT TTTCTCTGAA CTTGAAAGCT TAACAGATCC 3120
            Consensus   GGAGAAAATC CCTGGGAAGT TTAAGAAACT TTTCTCTGAA CTTGAAAGCT TAACAGATCC
    100%
Conservation
    0%
                                        3,140                          3,160                          3,180
RAPGEF5_WT_ mEGFP-N1    TTCCCTGAAT CACAAAGCCT ACAGAGATGC ATTCAAAAAG ATGAAACCAC CCAAAATCCC 3177
RAPGEF5_RISK_mEGFP-N1   TTCCCTGAAT CACAAAGCCT ACAGAGATGC ATTCAAAAAG ATGAAACCAC CCAAAATCCC 3180
            Consensus   TTCCCTGAAT CACAAAGCCT ACAGAGATGC ATTCAAAAAG ATGAAACCAC CCAAAATCCC
    100%
Conservation
    0%
                                        3,200                          3,220                          3,240
RAPGEF5_WT_ mEGFP-N1    ATTCATGCCT CTGTTGCTTA AAGATGTAAC ATTTATTCAT GAAGGAAATA AAACATTTTT 3237
RAPGEF5_RISK_mEGFP-N1   ATTCATGCCT CTGTTGCTTA AAGATGTAAC ATTTATTCAT GAAGGAAATA AAACATTTTT 3240
            Consensus   ATTCATGCCT CTGTTGCTTA AAGATGTAAC ATTTATTCAT GAAGGAAATA AAACATTTTT
    100%
Conservation
    0%
                                        3,260                          3,280                          3,300
RAPGEF5_WT_ mEGFP-N1    GGATAATCTT GTCAATTTTG AAAAACTGCA CATGATTGCA GACACCGTCC GGACCCTGAG 3297
RAPGEF5_RISK_mEGFP-N1   GGATAATCTT GTCAATTTTG AAAAACTGCA CATGATTGCA GACACCGTCC GGACCCTGAG 3300
            Consensus   GGATAATCTT GTCAATTTTG AAAAACTGCA CATGATTGCA GACACCGTCC GGACCCTGAG
    100%
Conservation
    0%
                                        3,320                          3,340                          3,360
RAPGEF5_WT_ mEGFP-N1    GCACTGCAGG ACCAATCAGT TCGGAAGCGA CGTGTCTCCA AAGGAGCACC AAGAGTTAAA 3357
RAPGEF5_RISK_mEGFP-N1   GCACTGCAGG ACCAATCAGT TCGGAAGCGA CGTGTCTCCA AAGGAGCACC AAGAGTTAAA 3360
            Consensus   GCACTGCAGG ACCAATCAGT TCGGAAGCGA CGTGTCTCCA AAGGAGCACC AAGAGTTAAA
    100%
Conservation
    0%
                                        3,380                          3,400                          3,420
RAPGEF5_WT_ mEGFP-N1    ATCCTATGTT AATCACCTGT ATGTCATTGA CAGCCAGCAG GCCTTGTTTG AGCTGTCACA 3417
RAPGEF5_RISK_mEGFP-N1   ATCCTATGTT AATCACCTGT ATGTCATTGA CAGCCAGCAG GCCTTGTTTG AGCTGTCACA 3420
            Consensus   ATCCTATGTT AATCACCTGT ATGTCATTGA CAGCCAGCAG GCCTTGTTTG AGCTGTCACA
    100%
Conservation
    0%
                                      * 3,440  BAMHI                   3,460                          3,480     RAPGEF5 gene end
RAPGEF5_WT_ mEGFP-N1    CAGGATCGAG CCTCGGGTGG GATCCACCGG TCGCCACCAT GGTGAGCAAG GGCGAGGAGC 3477
RAPGEF5_RISK_mEGFP-N1   CAGGATCGAG CCTCGGGTGG GATCCACCGG TCGCCACCAT GGTGAGCAAG GGCGAGGAGC 3480
            Consensus   CAGGATCGAG CCTCGGGTGG GATCCACCGG TCGCCACCAT GGTGAGCAAG GGCGAGGAGC
    100%
Conservation
    0%
                                        3,500                          3,520                          3,540
RAPGEF5_WT_ mEGFP-N1    TGTTCACCGG GGTGGTGCCC ATCCTGGTCG AGCTGGACGG CGACGTAAAC GGCCACAAGT 3537
RAPGEF5_RISK_mEGFP-N1   TGTTCACCGG GGTGGTGCCC ATCCTGGTCG AGCTGGACGG CGACGTAAAC GGCCACAAGT 3540
            Consensus   TGTTCACCGG GGTGGTGCCC ATCCTGGTCG AGCTGGACGG CGACGTAAAC GGCCACAAGT
    100%
Conservation
    0%
                                        3,560                          3,580                          3,600
RAPGEF5_WT_ mEGFP-N1    TCAGCGTGTC CGGCGAGGGC GAGGGCGATG CCACCTACGG CAAGCTGACC CTGAAGTTCA 3597
RAPGEF5_RISK_mEGFP-N1   TCAGCGTGTC CGGCGAGGGC GAGGGCGATG CCACCTACGG CAAGCTGACC CTGAAGTTCA 3600
            Consensus   TCAGCGTGTC CGGCGAGGGC GAGGGCGATG CCACCTACGG CAAGCTGACC CTGAAGTTCA
    100%
Conservation
    0%
```

```
                                         3,620                          3,640                          3,660
RAPGEF5_WT_ mEGFP-N1    TCTGCACCAC CGGCAAGCTG CCCGTGCCCT GGCCCACCCT CGTGACCACC CTGACCTACG 3657
RAPGEF5_RISK_mEGFP-N1   TCTGCACCAC CGGCAAGCTG CCCGTGCCCT GGCCCACCCT CGTGACCACC CTGACCTACG 3660
Consensus               TCTGCACCAC CGGCAAGCTG CCCGTGCCCT GGCCCACCCT CGTGACCACC CTGACCTACG
Conservation 100% 0%

                                         3,680                          3,700                          3,720
RAPGEF5_WT_ mEGFP-N1    GCGTGCAGTG CTTCAGCCGC TACCCCGACC ACATGAAGCA GCACGACTTC TTCAAGTCCG 3717
RAPGEF5_RISK_mEGFP-N1   GCGTGCAGTG CTTCAGCCGC TACCCCGACC ACATGAAGCA GCACGACTTC TTCAAGTCCG 3720
Consensus               GCGTGCAGTG CTTCAGCCGC TACCCCGACC ACATGAAGCA GCACGACTTC TTCAAGTCCG
Conservation 100% 0%

                                         3,740                          3,760                          3,780
RAPGEF5_WT_ mEGFP-N1    CCATGCCCGA AGGCTACGTC CAGGAGCGCA CCATCTTCTT CAAGGACGAC GGCAACTACA 3777
RAPGEF5_RISK_mEGFP-N1   CCATGCCCGA AGGCTACGTC CAGGAGCGCA CCATCTTCTT CAAGGACGAC GGCAACTACA 3780
Consensus               CCATGCCCGA AGGCTACGTC CAGGAGCGCA CCATCTTCTT CAAGGACGAC GGCAACTACA
Conservation 100% 0%

                                         3,800                          3,820                          3,840
RAPGEF5_WT_ mEGFP-N1    AGACCCGCGC CGAGGTGAAG TTCGAGGGCG ACACCCTGGT GAACCGCATC GAGCTGAAGG 3837
RAPGEF5_RISK_mEGFP-N1   AGACCCGCGC CGAGGTGAAG TTCGAGGGCG ACACCCTGGT GAACCGCATC GAGCTGAAGG 3840
Consensus               AGACCCGCGC CGAGGTGAAG TTCGAGGGCG ACACCCTGGT GAACCGCATC GAGCTGAAGG
Conservation 100% 0%

                                         3,860                          3,880                          3,900
RAPGEF5_WT_ mEGFP-N1    GCATCGACTT CAAGGAGGAC GGCAACATCC TGGGGCACAA GCTGGAGTAC AACTACAACA 3897
RAPGEF5_RISK_mEGFP-N1   GCATCGACTT CAAGGAGGAC GGCAACATCC TGGGGCACAA GCTGGAGTAC AACTACAACA 3900
Consensus               GCATCGACTT CAAGGAGGAC GGCAACATCC TGGGGCACAA GCTGGAGTAC AACTACAACA
Conservation 100% 0%

                                         3,920                          3,940                          3,960
RAPGEF5_WT_ mEGFP-N1    GCCACAACGT CTATATCATG GCCGACAAGC AGAAGAACGG CATCAAGGTG AACTTCAAGA 3957
RAPGEF5_RISK_mEGFP-N1   GCCACAACGT CTATATCATG GCCGACAAGC AGAAGAACGG CATCAAGGTG AACTTCAAGA 3960
Consensus               GCCACAACGT CTATATCATG GCCGACAAGC AGAAGAACGG CATCAAGGTG AACTTCAAGA
Conservation 100% 0%

                                         3,980                          4,000                          4,020
RAPGEF5_WT_ mEGFP-N1    TCCGCCACAA CATCGAGGAC GGCAGCGTGC AGCTCGCCGA CCACTACCAG CAGAACACCC 4017
RAPGEF5_RISK_mEGFP-N1   TCCGCCACAA CATCGAGGAC GGCAGCGTGC AGCTCGCCGA CCACTACCAG CAGAACACCC 4020
Consensus               TCCGCCACAA CATCGAGGAC GGCAGCGTGC AGCTCGCCGA CCACTACCAG CAGAACACCC
Conservation 100% 0%

                                         4,040                          4,060                          4,080
RAPGEF5_WT_ mEGFP-N1    CCATCGGCGA CGGCCCCGTG CTGCTGCCCG ACAACCACTA CCTGAGCACC CAGTCCAAGC 4077
RAPGEF5_RISK_mEGFP-N1   CCATCGGCGA CGGCCCCGTG CTGCTGCCCG ACAACCACTA CCTGAGCACC CAGTCCAAGC 4080
Consensus               CCATCGGCGA CGGCCCCGTG CTGCTGCCCG ACAACCACTA CCTGAGCACC CAGTCCAAGC
Conservation 100% 0%

                                         4,100                          4,120                          4,140
RAPGEF5_WT_ mEGFP-N1    TGAGCAAAGA CCCCAACGAG AAGCGCGATC ACATGGTCCT GCTGGAGTTC GTGACCGCCG 4137
RAPGEF5_RISK_mEGFP-N1   TGAGCAAAGA CCCCAACGAG AAGCGCGATC ACATGGTCCT GCTGGAGTTC GTGACCGCCG 4140
Consensus               TGAGCAAAGA CCCCAACGAG AAGCGCGATC ACATGGTCCT GCTGGAGTTC GTGACCGCCG
Conservation 100% 0%

                                         4,160                          4,180                          4,200
RAPGEF5_WT_ mEGFP-N1    CCGGGATCAC TCTCGGCATG GACGAGCTGT ACAAGTAAAG CGGCCGCGAC TCTAGATCAT 4197
RAPGEF5_RISK_mEGFP-N1   CCGGGATCAC TCTCGGCATG GACGAGCTGT ACAAGTAAAG CGGCCGCGAC TCTAGATCAT 4200
Consensus               CCGGGATCAC TCTCGGCATG GACGAGCTGT ACAAGTAAAG CGGCCGCGAC TCTAGATCAT
Conservation 100% 0%

                                         4,220                          4,240                          4,260
RAPGEF5_WT_ mEGFP-N1    AATCAGCCAT ACCACATTTG TAGAGGTTTT ACTTGCTTTA AAAAACCTCC CACACCTCCC 4257
RAPGEF5_RISK_mEGFP-N1   AATCAGCCAT ACCACATTTG TAGAGGTTTT ACTTGCTTTA AAAAACCTCC CACACCTCCC 4260
Consensus               AATCAGCCAT ACCACATTTG TAGAGGTTTT ACTTGCTTTA AAAAACCTCC CACACCTCCC
Conservation 100% 0%

                                         4,280                          4,300                          4,320
RAPGEF5_WT_ mEGFP-N1    CCTGAACCTG AAACATAAAA TGAATGCAAT TGTTGTTGTT AACTTGTTTA TTGCAGCTTA 4317
RAPGEF5_RISK_mEGFP-N1   CCTGAACCTG AAACATAAAA TGAATGCAAT TGTTGTTGTT AACTTGTTTA TTGCAGCTTA 4320
Consensus               CCTGAACCTG AAACATAAAA TGAATGCAAT TGTTGTTGTT AACTTGTTTA TTGCAGCTTA
Conservation 100% 0%
```

```
                                     4,340                   4,360                   4,380
RAPGEF5_WT_ mEGFP-N1    TAATGGTTAC AAATAAAGCA ATAGCATCAC AAATTTCACA AATAAAGCAT TTTTTTCACT 4377
RAPGEF5_RISK_mEGFP-N1   TAATGGTTAC AAATAAAGCA ATAGCATCAC AAATTTCACA AATAAAGCAT TTTTTTCACT 4380
Consensus               TAATGGTTAC AAATAAAGCA ATAGCATCAC AAATTTCACA AATAAAGCAT TTTTTTCACT
Conservation (100% / 0%)

                                     4,400                   4,420                   4,440
RAPGEF5_WT_ mEGFP-N1    GCATTCTAGT TGTGGTTTGT CCAAACTCAT CAATGTATCT TAAGGCGTAA ATTGTAAGCG 4437
RAPGEF5_RISK_mEGFP-N1   GCATTCTAGT TGTGGTTTGT CCAAACTCAT CAATGTATCT TAAGGCGTAA ATTGTAAGCG 4440
Consensus               GCATTCTAGT TGTGGTTTGT CCAAACTCAT CAATGTATCT TAAGGCGTAA ATTGTAAGCG
Conservation (100% / 0%)

                                     4,460                   4,480                   4,500
RAPGEF5_WT_ mEGFP-N1    TTAATATTTT GTTAAAATTC GCGTTAAATT TTTGTTAAAT CAGCTCATTT TTTAACCAAT 4497
RAPGEF5_RISK_mEGFP-N1   TTAATATTTT GTTAAAATTC GCGTTAAATT TTTGTTAAAT CAGCTCATTT TTTAACCAAT 4500
Consensus               TTAATATTTT GTTAAAATTC GCGTTAAATT TTTGTTAAAT CAGCTCATTT TTTAACCAAT
Conservation (100% / 0%)

                                     4,520                   4,540                   4,560
RAPGEF5_WT_ mEGFP-N1    AGGCCGAAAT CGGCAAAATC CCTTATAAAT CAAAAGAATA GACCGAGATA GGGTTGAGTG 4557
RAPGEF5_RISK_mEGFP-N1   AGGCCGAAAT CGGCAAAATC CCTTATAAAT CAAAAGAATA GACCGAGATA GGGTTGAGTG 4560
Consensus               AGGCCGAAAT CGGCAAAATC CCTTATAAAT CAAAAGAATA GACCGAGATA GGGTTGAGTG
Conservation (100% / 0%)

                                     4,580                   4,600                   4,620
RAPGEF5_WT_ mEGFP-N1    TTGTTCCAGT TTGGAACAAG AGTCCACTAT TAAAGAACGT GGACTCCAAC GTCAAAGGGC 4617
RAPGEF5_RISK_mEGFP-N1   TTGTTCCAGT TTGGAACAAG AGTCCACTAT TAAAGAACGT GGACTCCAAC GTCAAAGGGC 4620
Consensus               TTGTTCCAGT TTGGAACAAG AGTCCACTAT TAAAGAACGT GGACTCCAAC GTCAAAGGGC
Conservation (100% / 0%)

                                     4,640                   4,660                   4,680
RAPGEF5_WT_ mEGFP-N1    GAAAAACCGT CTATCAGGGC GATGGCCCAC TACGTGAACC ATCACCCTAA TCAAGTTTTT 4677
RAPGEF5_RISK_mEGFP-N1   GAAAAACCGT CTATCAGGGC GATGGCCCAC TACGTGAACC ATCACCCTAA TCAAGTTTTT 4680
Consensus               GAAAAACCGT CTATCAGGGC GATGGCCCAC TACGTGAACC ATCACCCTAA TCAAGTTTTT
Conservation (100% / 0%)

                                     4,700                   4,720                   4,740
RAPGEF5_WT_ mEGFP-N1    TGGGGTCGAG GTGCCGTAAA GCACTAAATC GGAACCCTAA AGGGAGCCCC CGATTTAGAG 4737
RAPGEF5_RISK_mEGFP-N1   TGGGGTCGAG GTGCCGTAAA GCACTAAATC GGAACCCTAA AGGGAGCCCC CGATTTAGAG 4740
Consensus               TGGGGTCGAG GTGCCGTAAA GCACTAAATC GGAACCCTAA AGGGAGCCCC CGATTTAGAG
Conservation (100% / 0%)

                                     4,760                   4,780                   4,800
RAPGEF5_WT_ mEGFP-N1    CTTGACGGGG AAAGCCGGCG AACGTGGCGA GAAAGGAAGG GAAGAAAGCG AAAGGAGCGG 4797
RAPGEF5_RISK_mEGFP-N1   CTTGACGGGG AAAGCCGGCG AACGTGGCGA GAAAGGAAGG GAAGAAAGCG AAAGGAGCGG 4800
Consensus               CTTGACGGGG AAAGCCGGCG AACGTGGCGA GAAAGGAAGG GAAGAAAGCG AAAGGAGCGG
Conservation (100% / 0%)

                                     4,820                   4,840                   4,860
RAPGEF5_WT_ mEGFP-N1    GCGCTAGGGC GCTGGCAAGT GTAGCGGTCA CGCTGCGCGT AACCACCACA CCCGCCGCGC 4857
RAPGEF5_RISK_mEGFP-N1   GCGCTAGGGC GCTGGCAAGT GTAGCGGTCA CGCTGCGCGT AACCACCACA CCCGCCGCGC 4860
Consensus               GCGCTAGGGC GCTGGCAAGT GTAGCGGTCA CGCTGCGCGT AACCACCACA CCCGCCGCGC
Conservation (100% / 0%)

                                     4,880                   4,900                   4,920
RAPGEF5_WT_ mEGFP-N1    TTAATGCGCC GCTACAGGGC GCGTCAGGTG GCACTTTTCG GGGAAATGTG CGCGGAACCC 4917
RAPGEF5_RISK_mEGFP-N1   TTAATGCGCC GCTACAGGGC GCGTCAGGTG GCACTTTTCG GGGAAATGTG CGCGGAACCC 4920
Consensus               TTAATGCGCC GCTACAGGGC GCGTCAGGTG GCACTTTTCG GGGAAATGTG CGCGGAACCC
Conservation (100% / 0%)

                                     4,940                   4,960                   4,980
RAPGEF5_WT_ mEGFP-N1    CTATTTGTTT ATTTTTCTAA ATACATTCAA ATATGTATCC GCTCATGAGA CAATAACCCT 4977
RAPGEF5_RISK_mEGFP-N1   CTATTTGTTT ATTTTTCTAA ATACATTCAA ATATGTATCC GCTCATGAGA CAATAACCCT 4980
Consensus               CTATTTGTTT ATTTTTCTAA ATACATTCAA ATATGTATCC GCTCATGAGA CAATAACCCT
Conservation (100% / 0%)

                                     5,000                   5,020                   5,040
RAPGEF5_WT_ mEGFP-N1    GATAAATGCT TCAATAATAT TGAAAAAGGA AGAGTCCTGA GGCGGAAAGA ACCAGCTGTG 5037
RAPGEF5_RISK_mEGFP-N1   GATAAATGCT TCAATAATAT TGAAAAAGGA AGAGTCCTGA GGCGGAAAGA ACCAGCTGTG 5040
Consensus               GATAAATGCT TCAATAATAT TGAAAAAGGA AGAGTCCTGA GGCGGAAAGA ACCAGCTGTG
Conservation (100% / 0%)
```

```
                                5,060                         5,080                         5,100
RAPGEF5_WT_ mEGFP-N1    GAATGTGTGT CAGTTAGGGT GTGGAAAGTC CCCAGGCTCC CCAGCAGGCA GAAGTATGCA 5097
RAPGEF5_RISK_mEGFP-N1   GAATGTGTGT CAGTTAGGGT GTGGAAAGTC CCCAGGCTCC CCAGCAGGCA GAAGTATGCA 5100
Consensus               GAATGTGTGT CAGTTAGGGT GTGGAAAGTC CCCAGGCTCC CCAGCAGGCA GAAGTATGCA
Conservation (100%–0%)

                                5,120                         5,140                         5,160
RAPGEF5_WT_ mEGFP-N1    AAGCATGCAT CTCAATTAGT CAGCAACCAG GTGTGGAAAG TCCCCAGGCT CCCCAGCAGG 5157
RAPGEF5_RISK_mEGFP-N1   AAGCATGCAT CTCAATTAGT CAGCAACCAG GTGTGGAAAG TCCCCAGGCT CCCCAGCAGG 5160
Consensus               AAGCATGCAT CTCAATTAGT CAGCAACCAG GTGTGGAAAG TCCCCAGGCT CCCCAGCAGG
Conservation (100%–0%)

                                5,180                         5,200                         5,220
RAPGEF5_WT_ mEGFP-N1    CAGAAGTATG CAAAGCATGC ATCTCAATTA GTCAGCAACC ATAGTCCCGC CCCTAACTCC 5217
RAPGEF5_RISK_mEGFP-N1   CAGAAGTATG CAAAGCATGC ATCTCAATTA GTCAGCAACC ATAGTCCCGC CCCTAACTCC 5220
Consensus               CAGAAGTATG CAAAGCATGC ATCTCAATTA GTCAGCAACC ATAGTCCCGC CCCTAACTCC
Conservation (100%–0%)

                                5,240                         5,260                         5,280
RAPGEF5_WT_ mEGFP-N1    GCCCATCCCG CCCCTAACTC CGCCCAGTTC CGCCCATTCT CCGCCCCATG GCTGACTAAT 5277
RAPGEF5_RISK_mEGFP-N1   GCCCATCCCG CCCCTAACTC CGCCCAGTTC CGCCCATTCT CCGCCCCATG GCTGACTAAT 5280
Consensus               GCCCATCCCG CCCCTAACTC CGCCCAGTTC CGCCCATTCT CCGCCCCATG GCTGACTAAT
Conservation (100%–0%)

                                5,300                         5,320                         5,340
RAPGEF5_WT_ mEGFP-N1    TTTTTTTATT TATGCAGAGG CCGAGGCCGC CTCGGCCTCT GAGCTATTCC AGAAGTAGTG 5337
RAPGEF5_RISK_mEGFP-N1   TTTTTTTATT TATGCAGAGG CCGAGGCCGC CTCGGCCTCT GAGCTATTCC AGAAGTAGTG 5340
Consensus               TTTTTTTATT TATGCAGAGG CCGAGGCCGC CTCGGCCTCT GAGCTATTCC AGAAGTAGTG
Conservation (100%–0%)

                                5,360                         5,380                         5,400
RAPGEF5_WT_ mEGFP-N1    AGGAGGCTTT TTTGGAGGCC TAGGCTTTTG CAAAGATCGA TCAAGAGACA GGATGAGGAT 5397
RAPGEF5_RISK_mEGFP-N1   AGGAGGCTTT TTTGGAGGCC TAGGCTTTTG CAAAGATCGA TCAAGAGACA GGATGAGGAT 5400
Consensus               AGGAGGCTTT TTTGGAGGCC TAGGCTTTTG CAAAGATCGA TCAAGAGACA GGATGAGGAT
Conservation (100%–0%)

                                5,420                         5,440                         5,460
RAPGEF5_WT_ mEGFP-N1    CGTTTCGCAT GATTGAACAA GATGGATTGC ACGCAGGTTC TCCGGCCGCT TGGGTGGAGA 5457
RAPGEF5_RISK_mEGFP-N1   CGTTTCGCAT GATTGAACAA GATGGATTGC ACGCAGGTTC TCCGGCCGCT TGGGTGGAGA 5460
Consensus               CGTTTCGCAT GATTGAACAA GATGGATTGC ACGCAGGTTC TCCGGCCGCT TGGGTGGAGA
Conservation (100%–0%)

                                5,480                         5,500                         5,520
RAPGEF5_WT_ mEGFP-N1    GGCTATTCGG CTATGACTGG GCACAACAGA CAATCGGCTG CTCTGATGCC GCCGTGTTCC 5517
RAPGEF5_RISK_mEGFP-N1   GGCTATTCGG CTATGACTGG GCACAACAGA CAATCGGCTG CTCTGATGCC GCCGTGTTCC 5520
Consensus               GGCTATTCGG CTATGACTGG GCACAACAGA CAATCGGCTG CTCTGATGCC GCCGTGTTCC
Conservation (100%–0%)

                                5,540                         5,560                         5,580
RAPGEF5_WT_ mEGFP-N1    GGCTGTCAGC GCAGGGGCGC CCGGTTCTTT TTGTCAAGAC CGACCTGTCC GGTGCCCTGA 5577
RAPGEF5_RISK_mEGFP-N1   GGCTGTCAGC GCAGGGGCGC CCGGTTCTTT TTGTCAAGAC CGACCTGTCC GGTGCCCTGA 5580
Consensus               GGCTGTCAGC GCAGGGGCGC CCGGTTCTTT TTGTCAAGAC CGACCTGTCC GGTGCCCTGA
Conservation (100%–0%)

                                5,600                         5,620                         5,640
RAPGEF5_WT_ mEGFP-N1    ATGAACTGCA AGACGAGGCA GCGCGGCTAT CGTGGCTGGC CACGACGGGC GTTCCTTGCG 5637
RAPGEF5_RISK_mEGFP-N1   ATGAACTGCA AGACGAGGCA GCGCGGCTAT CGTGGCTGGC CACGACGGGC GTTCCTTGCG 5640
Consensus               ATGAACTGCA AGACGAGGCA GCGCGGCTAT CGTGGCTGGC CACGACGGGC GTTCCTTGCG
Conservation (100%–0%)

                                5,660                         5,680                         5,700
RAPGEF5_WT_ mEGFP-N1    CAGCTGTGCT CGACGTTGTC ACTGAAGCGG GAAGGGACTG GCTGCTATTG GGCGAAGTGC 5697
RAPGEF5_RISK_mEGFP-N1   CAGCTGTGCT CGACGTTGTC ACTGAAGCGG GAAGGGACTG GCTGCTATTG GGCGAAGTGC 5700
Consensus               CAGCTGTGCT CGACGTTGTC ACTGAAGCGG GAAGGGACTG GCTGCTATTG GGCGAAGTGC
Conservation (100%–0%)

                                5,720                         5,740                         5,760
RAPGEF5_WT_ mEGFP-N1    CGGGGCAGGA TCTCCTGTCA TCTCACCTTG CTCCTGCCGA GAAAGTATCC ATCATGGCTG 5757
RAPGEF5_RISK_mEGFP-N1   CGGGGCAGGA TCTCCTGTCA TCTCACCTTG CTCCTGCCGA GAAAGTATCC ATCATGGCTG 5760
Consensus               CGGGGCAGGA TCTCCTGTCA TCTCACCTTG CTCCTGCCGA GAAAGTATCC ATCATGGCTG
Conservation (100%–0%)
```

```
                                                5,780                       5,800                       5,820
RAPGEF5_WT_ mEGFP-N1    ATGCAATGCG GCGGCTGCAT ACGCTTGATC CGGCTACCTG CCCATTCGAC CACCAAGCGA 5817
RAPGEF5_RISK_mEGFP-N1   ATGCAATGCG GCGGCTGCAT ACGCTTGATC CGGCTACCTG CCCATTCGAC CACCAAGCGA 5820
Consensus               ATGCAATGCG GCGGCTGCAT ACGCTTGATC CGGCTACCTG CCCATTCGAC CACCAAGCGA
Conservation 100% / 0%

                                                5,840                       5,860                       5,880
RAPGEF5_WT_ mEGFP-N1    AACATCGCAT CGAGCGAGCA CGTACTCGGA TGGAAGCCGG TCTTGTCGAT CAGGATGATC 5877
RAPGEF5_RISK_mEGFP-N1   AACATCGCAT CGAGCGAGCA CGTACTCGGA TGGAAGCCGG TCTTGTCGAT CAGGATGATC 5880
Consensus               AACATCGCAT CGAGCGAGCA CGTACTCGGA TGGAAGCCGG TCTTGTCGAT CAGGATGATC
Conservation 100% / 0%

                                                5,900                       5,920                       5,940
RAPGEF5_WT_ mEGFP-N1    TGGACGAAGA GCATCAGGGG CTCGCGCCAG CCGAACTGTT CGCCAGGCTC AAGGCGAGCA 5937
RAPGEF5_RISK_mEGFP-N1   TGGACGAAGA GCATCAGGGG CTCGCGCCAG CCGAACTGTT CGCCAGGCTC AAGGCGAGCA 5940
Consensus               TGGACGAAGA GCATCAGGGG CTCGCGCCAG CCGAACTGTT CGCCAGGCTC AAGGCGAGCA
Conservation 100% / 0%

                                                5,960                       5,980                       6,000
RAPGEF5_WT_ mEGFP-N1    TGCCCGACGG CGAGGATCTC GTCGTGACCC ATGGCGATGC CTGCTTGCCG AATATCATGG 5997
RAPGEF5_RISK_mEGFP-N1   TGCCCGACGG CGAGGATCTC GTCGTGACCC ATGGCGATGC CTGCTTGCCG AATATCATGG 6000
Consensus               TGCCCGACGG CGAGGATCTC GTCGTGACCC ATGGCGATGC CTGCTTGCCG AATATCATGG
Conservation 100% / 0%

                                                6,020                       6,040                       6,060
RAPGEF5_WT_ mEGFP-N1    TGGAAAATGG CCGCTTTTCT GGATTCATCG ACTGTGGCCG GCTGGGTGTG GCGGACCGCT 6057
RAPGEF5_RISK_mEGFP-N1   TGGAAAATGG CCGCTTTTCT GGATTCATCG ACTGTGGCCG GCTGGGTGTG GCGGACCGCT 6060
Consensus               TGGAAAATGG CCGCTTTTCT GGATTCATCG ACTGTGGCCG GCTGGGTGTG GCGGACCGCT
Conservation 100% / 0%

                                                6,080                       6,100                       6,120
RAPGEF5_WT_ mEGFP-N1    ATCAGGACAT AGCGTTGGCT ACCCGTGATA TTGCTGAAGA GCTTGGCGGC GAATGGGCTG 6117
RAPGEF5_RISK_mEGFP-N1   ATCAGGACAT AGCGTTGGCT ACCCGTGATA TTGCTGAAGA GCTTGGCGGC GAATGGGCTG 6120
Consensus               ATCAGGACAT AGCGTTGGCT ACCCGTGATA TTGCTGAAGA GCTTGGCGGC GAATGGGCTG
Conservation 100% / 0%

                                                6,140                       6,160                       6,180
RAPGEF5_WT_ mEGFP-N1    ACCGCTTCCT CGTGCTTTAC GGTATCGCCG CTCCCGATTC GCAGCGCATC GCCTTCTATC 6177
RAPGEF5_RISK_mEGFP-N1   ACCGCTTCCT CGTGCTTTAC GGTATCGCCG CTCCCGATTC GCAGCGCATC GCCTTCTATC 6180
Consensus               ACCGCTTCCT CGTGCTTTAC GGTATCGCCG CTCCCGATTC GCAGCGCATC GCCTTCTATC
Conservation 100% / 0%

                                                6,200                       6,220                       6,240
RAPGEF5_WT_ mEGFP-N1    GCCTTCTTGA CGAGTTCTTC TGAGCGGGAC TCTGGGGTTC GAAATGACCG ACCAAGCGAC 6237
RAPGEF5_RISK_mEGFP-N1   GCCTTCTTGA CGAGTTCTTC TGAGCGGGAC TCTGGGGTTC GAAATGACCG ACCAAGCGAC 6240
Consensus               GCCTTCTTGA CGAGTTCTTC TGAGCGGGAC TCTGGGGTTC GAAATGACCG ACCAAGCGAC
Conservation 100% / 0%

                                                6,260                       6,280                       6,300
RAPGEF5_WT_ mEGFP-N1    GCCCAACCTG CCATCACGAG ATTTCGATTC CACCGCCGCC TTCTATGAAA GGTTGGGCTT 6297
RAPGEF5_RISK_mEGFP-N1   GCCCAACCTG CCATCACGAG ATTTCGATTC CACCGCCGCC TTCTATGAAA GGTTGGGCTT 6300
Consensus               GCCCAACCTG CCATCACGAG ATTTCGATTC CACCGCCGCC TTCTATGAAA GGTTGGGCTT
Conservation 100% / 0%

                                                6,320                       6,340                       6,360
RAPGEF5_WT_ mEGFP-N1    CGGAATCGTT TTCCGGGACG CCGGCTGGAT GATCCTCCAG CGCGGGGATC TCATGCTGGA 6357
RAPGEF5_RISK_mEGFP-N1   CGGAATCGTT TTCCGGGACG CCGGCTGGAT GATCCTCCAG CGCGGGGATC TCATGCTGGA 6360
Consensus               CGGAATCGTT TTCCGGGACG CCGGCTGGAT GATCCTCCAG CGCGGGGATC TCATGCTGGA
Conservation 100% / 0%

                                                6,380                       6,400                       6,420
RAPGEF5_WT_ mEGFP-N1    GTTCTTCGCC CACCCTAGGG GGAGGCTAAC TGAAACACGG AAGGAGACAA TACCGGAAGG 6417
RAPGEF5_RISK_mEGFP-N1   GTTCTTCGCC CACCCTAGGG GGAGGCTAAC TGAAACACGG AAGGAGACAA TACCGGAAGG 6420
Consensus               GTTCTTCGCC CACCCTAGGG GGAGGCTAAC TGAAACACGG AAGGAGACAA TACCGGAAGG
Conservation 100% / 0%

                                                6,440                       6,460                       6,480
RAPGEF5_WT_ mEGFP-N1    AACCCGCGCT ATGACGGCAA TAAAAAGACA GAATAAAACG CACGGTGTTG GGTCGTTTGT 6477
RAPGEF5_RISK_mEGFP-N1   AACCCGCGCT ATGACGGCAA TAAAAAGACA GAATAAAACG CACGGTGTTG GGTCGTTTGT 6480
Consensus               AACCCGCGCT ATGACGGCAA TAAAAAGACA GAATAAAACG CACGGTGTTG GGTCGTTTGT
Conservation 100% / 0%
```

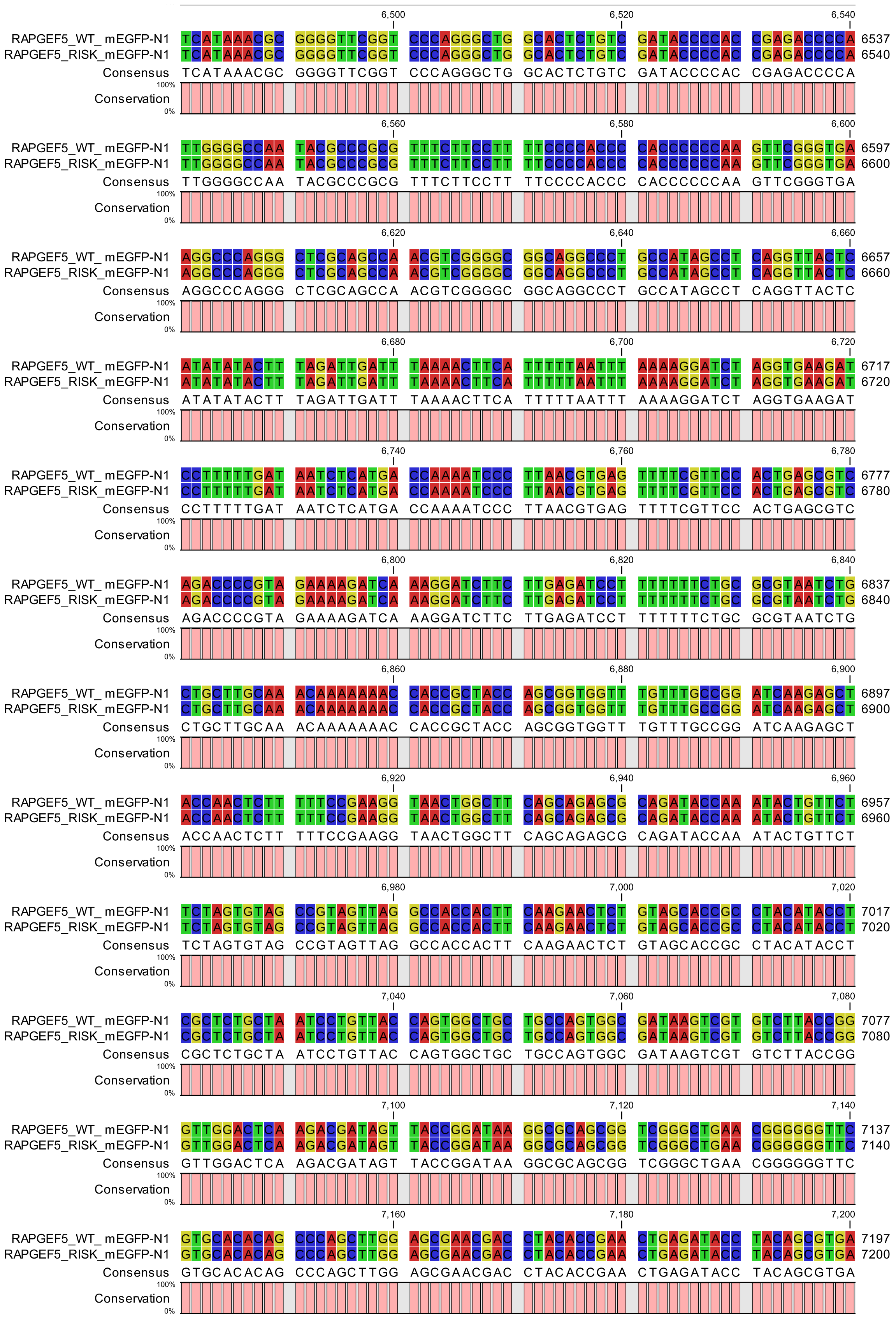
6,500 6,520 6,540
RAPGEF5_WT_ mEGFP-N1 TCATAAACGC GGGGTTCGGT CCCAGGGCTG GCACTCTGTC GATACCCCAC CGAGACCCCA 6537
RAPGEF5_RISK_mEGFP-N1 TCATAAACGC GGGGTTCGGT CCCAGGGCTG GCACTCTGTC GATACCCCAC CGAGACCCCA 6540
Consensus TCATAAACGC GGGGTTCGGT CCCAGGGCTG GCACTCTGTC GATACCCCAC CGAGACCCCA
100% Conservation 0%
6,560 6,580 6,600
RAPGEF5_WT_ mEGFP-N1 TTGGGGCCAA TACGCCCGCG TTTCTTCCTT TTCCCCACCC CACCCCCCAA GTTCGGGTGA 6597
RAPGEF5_RISK_mEGFP-N1 TTGGGGCCAA TACGCCCGCG TTTCTTCCTT TTCCCCACCC CACCCCCCAA GTTCGGGTGA 6600
Consensus TTGGGGCCAA TACGCCCGCG TTTCTTCCTT TTCCCCACCC CACCCCCCAA GTTCGGGTGA
100% Conservation 0%
6,620 6,640 6,660
RAPGEF5_WT_ mEGFP-N1 AGGCCCAGGG CTCGCAGCCA ACGTCGGGGC GGCAGGCCCT GCCATAGCCT CAGGTTACTC 6657
RAPGEF5_RISK_mEGFP-N1 AGGCCCAGGG CTCGCAGCCA ACGTCGGGGC GGCAGGCCCT GCCATAGCCT CAGGTTACTC 6660
Consensus AGGCCCAGGG CTCGCAGCCA ACGTCGGGGC GGCAGGCCCT GCCATAGCCT CAGGTTACTC
100% Conservation 0%
6,680 6,700 6,720
RAPGEF5_WT_ mEGFP-N1 ATATATACTT TAGATTGATT TAAAACTTCA TTTTTAATTT AAAAGGATCT AGGTGAAGAT 6717
RAPGEF5_RISK_mEGFP-N1 ATATATACTT TAGATTGATT TAAAACTTCA TTTTTAATTT AAAAGGATCT AGGTGAAGAT 6720
Consensus ATATATACTT TAGATTGATT TAAAACTTCA TTTTTAATTT AAAAGGATCT AGGTGAAGAT
100% Conservation 0%
6,740 6,760 6,780
RAPGEF5_WT_ mEGFP-N1 CCTTTTTGAT AATCTCATGA CCAAAATCCC TTAACGTGAG TTTTCGTTCC ACTGAGCGTC 6777
RAPGEF5_RISK_mEGFP-N1 CCTTTTTGAT AATCTCATGA CCAAAATCCC TTAACGTGAG TTTTCGTTCC ACTGAGCGTC 6780
Consensus CCTTTTTGAT AATCTCATGA CCAAAATCCC TTAACGTGAG TTTTCGTTCC ACTGAGCGTC
100% Conservation 0%
6,800 6,820 6,840
RAPGEF5_WT_ mEGFP-N1 AGACCCCGTA GAAAAGATCA AAGGATCTTC TTGAGATCCT TTTTTTCTGC GCGTAATCTG 6837
RAPGEF5_RISK_mEGFP-N1 AGACCCCGTA GAAAAGATCA AAGGATCTTC TTGAGATCCT TTTTTTCTGC GCGTAATCTG 6840
Consensus AGACCCCGTA GAAAAGATCA AAGGATCTTC TTGAGATCCT TTTTTTCTGC GCGTAATCTG
100% Conservation 0%
6,860 6,880 6,900
RAPGEF5_WT_ mEGFP-N1 CTGCTTGCAA ACAAAAAAAC CACCGCTACC AGCGGTGGTT TGTTTGCCGG ATCAAGAGCT 6897
RAPGEF5_RISK_mEGFP-N1 CTGCTTGCAA ACAAAAAAAC CACCGCTACC AGCGGTGGTT TGTTTGCCGG ATCAAGAGCT 6900
Consensus CTGCTTGCAA ACAAAAAAAC CACCGCTACC AGCGGTGGTT TGTTTGCCGG ATCAAGAGCT
100% Conservation 0%
6,920 6,940 6,960
RAPGEF5_WT_ mEGFP-N1 ACCAACTCTT TTTCCGAAGG TAACTGGCTT CAGCAGAGCG CAGATACCAA ATACTGTTCT 6957
RAPGEF5_RISK_mEGFP-N1 ACCAACTCTT TTTCCGAAGG TAACTGGCTT CAGCAGAGCG CAGATACCAA ATACTGTTCT 6960
Consensus ACCAACTCTT TTTCCGAAGG TAACTGGCTT CAGCAGAGCG CAGATACCAA ATACTGTTCT
100% Conservation 0%
6,980 7,000 7,020
RAPGEF5_WT_ mEGFP-N1 TCTAGTGTAG CCGTAGTTAG GCCACCACTT CAAGAACTCT GTAGCACCGC CTACATACCT 7017
RAPGEF5_RISK_mEGFP-N1 TCTAGTGTAG CCGTAGTTAG GCCACCACTT CAAGAACTCT GTAGCACCGC CTACATACCT 7020
Consensus TCTAGTGTAG CCGTAGTTAG GCCACCACTT CAAGAACTCT GTAGCACCGC CTACATACCT
100% Conservation 0%
7,040 7,060 7,080
RAPGEF5_WT_ mEGFP-N1 CGCTCTGCTA ATCCTGTTAC CAGTGGCTGC TGCCAGTGGC GATAAGTCGT GTCTTACCGG 7077
RAPGEF5_RISK_mEGFP-N1 CGCTCTGCTA ATCCTGTTAC CAGTGGCTGC TGCCAGTGGC GATAAGTCGT GTCTTACCGG 7080
Consensus CGCTCTGCTA ATCCTGTTAC CAGTGGCTGC TGCCAGTGGC GATAAGTCGT GTCTTACCGG
100% Conservation 0%
7,100 7,120 7,140
RAPGEF5_WT_ mEGFP-N1 GTTGGACTCA AGACGATAGT TACCGGATAA GGCGCAGCGG TCGGGCTGAA CGGGGGGTTC 7137
RAPGEF5_RISK_mEGFP-N1 GTTGGACTCA AGACGATAGT TACCGGATAA GGCGCAGCGG TCGGGCTGAA CGGGGGGTTC 7140
Consensus GTTGGACTCA AGACGATAGT TACCGGATAA GGCGCAGCGG TCGGGCTGAA CGGGGGGTTC
100% Conservation 0%
7,160 7,180 7,200
RAPGEF5_WT_ mEGFP-N1 GTGCACACAG CCCAGCTTGG AGCGAACGAC CTACACCGAA CTGAGATACC TACAGCGTGA 7197
RAPGEF5_RISK_mEGFP-N1 GTGCACACAG CCCAGCTTGG AGCGAACGAC CTACACCGAA CTGAGATACC TACAGCGTGA 7200
Consensus GTGCACACAG CCCAGCTTGG AGCGAACGAC CTACACCGAA CTGAGATACC TACAGCGTGA
100% Conservation 0%

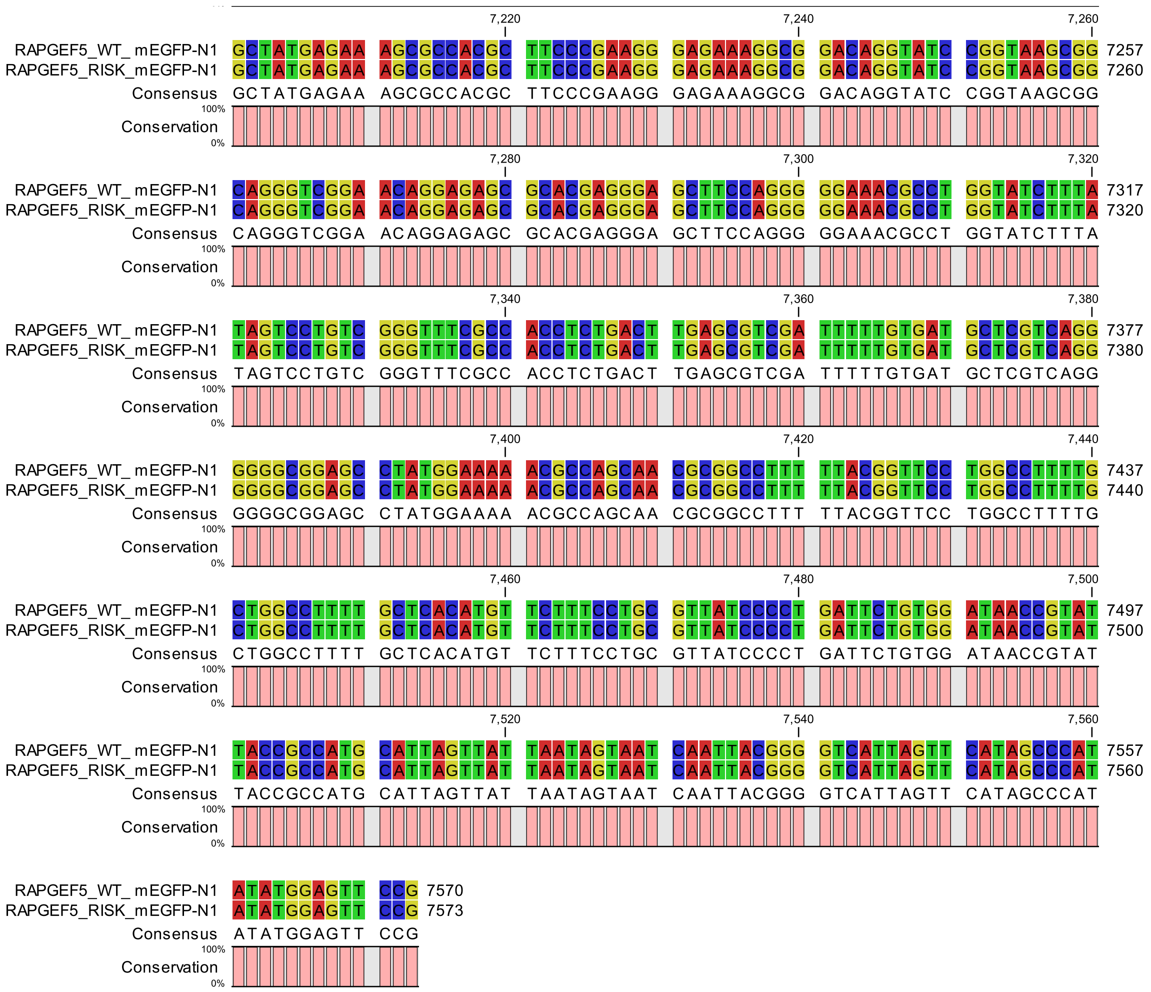
7,220
7,240
7,260
RAPGEF5_WT_ mEGFP-N1 GCTATGAGAA AGCGCCACGC TTCCCGAAGG GAGAAAGGCG GACAGGTATC CGGTAAGCGG 7257
RAPGEF5_RISK_mEGFP-N1 GCTATGAGAA AGCGCCACGC TTCCCGAAGG GAGAAAGGCG GACAGGTATC CGGTAAGCGG 7260
Consensus GCTATGAGAA AGCGCCACGC TTCCCGAAGG GAGAAAGGCG GACAGGTATC CGGTAAGCGG
100%
Conservation
0%
7,280
7,300
7,320
RAPGEF5_WT_ mEGFP-N1 CAGGGTCGGA ACAGGAGAGC GCACGAGGGA GCTTCCAGGG GGAAACGCCT GGTATCTTTA 7317
RAPGEF5_RISK_mEGFP-N1 CAGGGTCGGA ACAGGAGAGC GCACGAGGGA GCTTCCAGGG GGAAACGCCT GGTATCTTTA 7320
Consensus CAGGGTCGGA ACAGGAGAGC GCACGAGGGA GCTTCCAGGG GGAAACGCCT GGTATCTTTA
100%
Conservation
0%
7,340
7,360
7,380
RAPGEF5_WT_ mEGFP-N1 TAGTCCTGTC GGGTTTCGCC ACCTCTGACT TGAGCGTCGA TTTTTGTGAT GCTCGTCAGG 7377
RAPGEF5_RISK_mEGFP-N1 TAGTCCTGTC GGGTTTCGCC ACCTCTGACT TGAGCGTCGA TTTTTGTGAT GCTCGTCAGG 7380
Consensus TAGTCCTGTC GGGTTTCGCC ACCTCTGACT TGAGCGTCGA TTTTTGTGAT GCTCGTCAGG
100%
Conservation
0%
7,400
7,420
7,440
RAPGEF5_WT_ mEGFP-N1 GGGGCGGAGC CTATGGAAAA ACGCCAGCAA CGCGGCCTTT TTACGGTTCC TGGCCTTTTG 7437
RAPGEF5_RISK_mEGFP-N1 GGGGCGGAGC CTATGGAAAA ACGCCAGCAA CGCGGCCTTT TTACGGTTCC TGGCCTTTTG 7440
Consensus GGGGCGGAGC CTATGGAAAA ACGCCAGCAA CGCGGCCTTT TTACGGTTCC TGGCCTTTTG
100%
Conservation
0%
7,460
7,480
7,500
RAPGEF5_WT_ mEGFP-N1 CTGGCCTTTT GCTCACATGT TCTTTCCTGC GTTATCCCCT GATTCTGTGG ATAACCGTAT 7497
RAPGEF5_RISK_mEGFP-N1 CTGGCCTTTT GCTCACATGT TCTTTCCTGC GTTATCCCCT GATTCTGTGG ATAACCGTAT 7500
Consensus CTGGCCTTTT GCTCACATGT TCTTTCCTGC GTTATCCCCT GATTCTGTGG ATAACCGTAT
100%
Conservation
0%
7,520
7,540
7,560
RAPGEF5_WT_ mEGFP-N1 TACCGCCATG CATTAGTTAT TAATAGTAAT CAATTACGGG GTCATTAGTT CATAGCCCAT 7557
RAPGEF5_RISK_mEGFP-N1 TACCGCCATG CATTAGTTAT TAATAGTAAT CAATTACGGG GTCATTAGTT CATAGCCCAT 7560
Consensus TACCGCCATG CATTAGTTAT TAATAGTAAT CAATTACGGG GTCATTAGTT CATAGCCCAT
100%
Conservation
0%
RAPGEF5_WT_ mEGFP-N1 ATATGGAGTT CCG 7570
RAPGEF5_RISK_mEGFP-N1 ATATGGAGTT CCG 7573
Consensus ATATGGAGTT CCG
100%
Conservation
0%

Additional file 2 Supplemental Fig. 2b

mEGFP-N1 Plasmid Map

Addgene https://www.addgene.org/54767/

mEGFP-N1 was a gift from Michael Davidson (Addgene plasmid # 54767; http://n2t.net/addgene:54767; RRID:Addgene_54767)

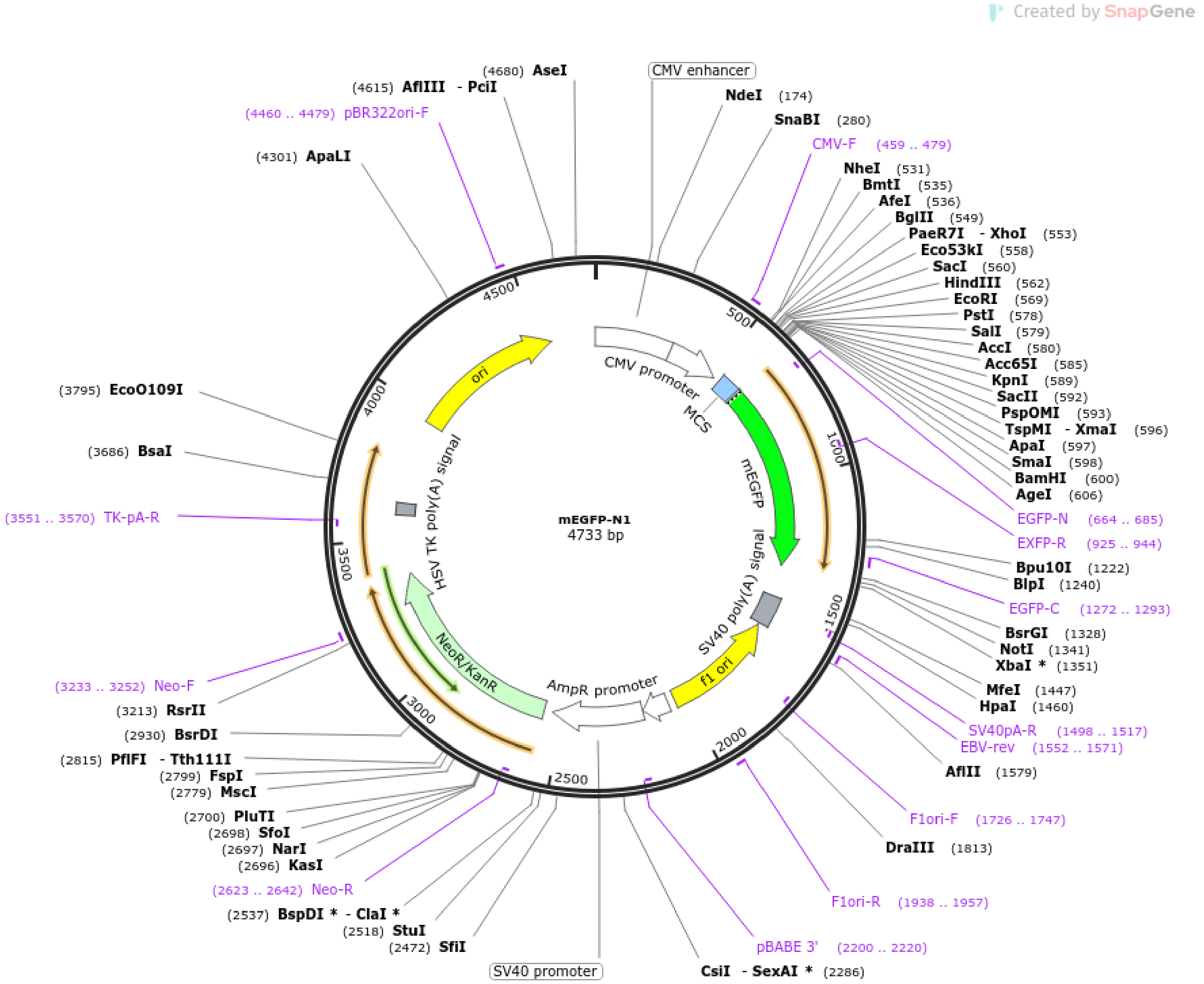

Supplement: Supplementary file 2 — Additional file 2: Supplemental Fig. 2a RAPGEF5 cDNA in mEGFP-N1 Expression Vector Wild Type (WT) and Risk Variant (RISK) Sequence Alignment. Supplemental Fig. 2b mEGFP-N1 Plasmid Map [file 40575_2024_138_MOESM2_ESM.pdf]
