# Supplementary figures and images for: Cellular localization of a variant RAPGEF5 protein associated with idiopathic epilepsy risk in the Belgian shepherd
Source: Canine Med Genet. 2024 Sep 29;11:4. doi: 10.1186/s40575-024-00138-3 (PMC11439299; doi:10.1186/s40575-024-00138-3)

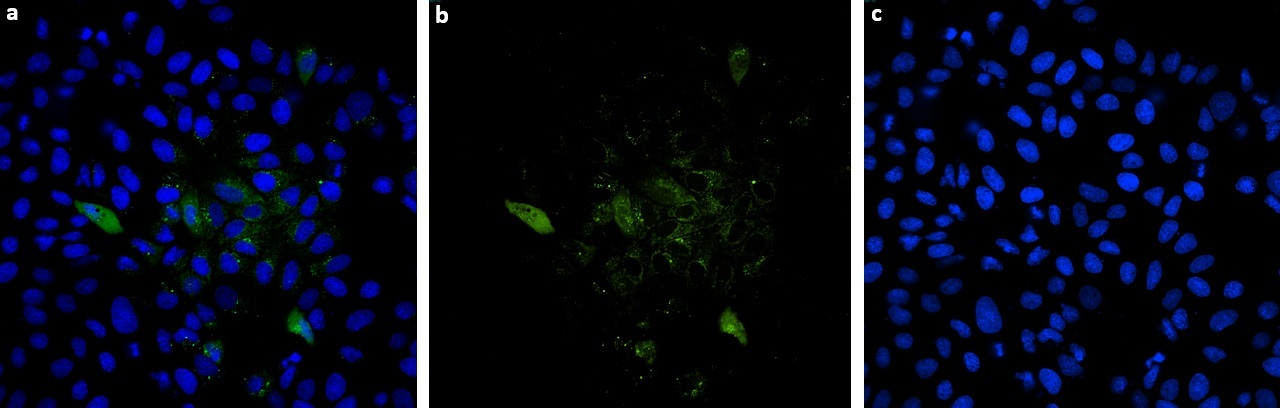

Supplement: Supplementary file 3 — Additional file 3: Supplemental Fig. 3 Confocal microscopic images of risk RAPGEF5-GFP fusion proteins in MDCK cells after transfection (40X magnification): (a) Overlay image of Hoechst 33342-stained nuclei and RAPGEF5-GFP fusion proteins; (b) RAPGEF5-GFP fusion proteins; (c) Hoechst 33342-stained nuclei [file 40575_2024_138_MOESM3_ESM.tif]
